# Traditional clinical symptoms and signs: Kampo pattern diagnosis in modern gastrointestinal disease

**DOI:** 10.3389/fphar.2024.1426491

**Published:** 2024-09-27

**Authors:** Paul Zedler, Judith Büntzel, Kenny Kuchta, Denichiro Yamaoka, Nanoha Sato, Kenji Watanabe, Silke Cameron

**Affiliations:** ^1^ Clinic for Gastroenterology and Gastrointestinal Oncology, University Medical Center Göttingen (UMG), Göttingen, Germany; ^2^ Department of Gastroenterology and General Internal Medicine, Clinic Hann. Münden, Hann. Münden, Germany; ^3^ Clinic for Haemato-Oncology, University Medical Center Göttingen (UMG), Göttingen, Germany; ^4^ Research Unit for East Asian Medicine, Department of Vegetation Analysis and Phytodiversity, Albrecht-von-Haller Institute, Göttingen, Germany; ^5^ Department for East Asian Traditional Medicine, Ehime Prefectural Central Hospital, Matsuyama, Japan; ^6^ Kampo Clinical Center, Hiroshima University, Hiroshima, Japan; ^7^ Yokohama University of Pharmacy, Yokohama, Japan

**Keywords:** Kampo medicine, Kampo pattern diagnosis, symptom questionnaire, body constitution, gastrointestinal disease

## Abstract

**Introduction:**

In traditional Japanese Kampo medicine, a profound anamnesis is completed by clinical examination. The resulting clinical image forms the basis of the patient’s diagnosis pattern, including the recent mental, physical, and social contexts. Kampo questionnaires support pattern diagnosis and bridge traditional and Western medicine diagnoses.

**Aims of the study:**

Traditional Kampo therapy is tailored to a specific body constitution, while Western medicine treatment is tailored to a specific disease. The aims of this study were to analyze whether traditional Kampo diagnosis is applicable to German patients and whether specific symptom patterns are characteristic for defined diseases.

**Material and methods:**

This study validates for the first time a Kampo questionnaire adapted for German patients. The analysis focuses on patients with gastrointestinal diseases, the main field for Kampo medicines.

**Results:**

In total, we prospectively included 251 participants; of those, 58 were cancer patients (23.1%), 35 had Crohn’s disease (13.9%), 18 had ulcerative colitis (7.2%), 17 had irritable bowel syndrome (6.8%), and 103 had other abdominal diseases (41%), as well as 20 German controls (8%). The patient population consisted of 144 female (57.4%) and 107 male (42.6%) participants. The median age was 65 years. The disease duration (average: 211 months) varied from 1 month (cancer patient) to 540 months (patient with Crohn’s disease). The scores for questions on the state of mind were significantly higher in patients with inflammatory bowel disease (IBD) as well as irritable bowel syndrome (IBS)—in comparison to the tumor and control groups. This was reflected in questions about abdominal discomfort, appetite, fecal habits, and cold sensation. Accordingly, symptoms of *Qi* (i.e., *vital energy*) deficiency were mostly observed in patients with chronic diseases such as Crohn’s disease and ulcerative colitis. Defined symptom combinations did not reflect conventional Western diagnosis.

**Conclusion:**

Our study results show that symptom patterns are independent of the underlying disease. They rather depict the individual patient within an individual time frame. Traditional Kampo questionnaires were found to be valid for the analysis of a patients’ body constitution (*sho*) and serve as a guide for Kampo treatment. We propose that individual pattern diagnosis should be taken into account to help treatment individualization.

## Introduction

Japanese phytotherapy, Kampo medicine, is a historically developed traditional medical system, originally based on ancient Chinese medicine compiled during the Han period (202 BC to 220 AD). This knowledge was introduced to Japan between the 6th and 8th centuries. From this common beginning, Japanese Kampo medicine developed its own concepts, and several authentic Japanese schools of Kampo developed (Kuchta, 2019). One of the later schools was the Koho-ha (school of classical formulas) (Kuchta, 2014a). For Yoshimasu Todo (1702–1773), its most prominent founder, the history of the patient (*monshin*/問診), inspection, and physical examination were the keys for understanding the body constitution (*sho*/証) and choosing the corresponding medication (*ho*/方) (Terasawa, 2004). The presenting symptoms or *symptom patterns* were the basis for the choice of medication (*hosho sotai*/方証相対) (Yakubo et al., 2014; Wu et al., 2021). Kampo patterns have recently been defined as “complete clinical presentation of the patient at a given moment in time” (WHO, 2019).

The body constitution can be determined from the patients’ history, i.e., age and sex, general health, pain sensation, temperature sensitivity, appetite, diet and digestion, urination, menstruation, vision, hearing, and sleep. To bridge the gap between ancient clinical understanding of a patients’ physiological situation and modern disease, questionnaires have been developed that cover areas including the patients’ current mental, physical, and social states. This anamnesis is complemented by a clinical examination including inspection, abdominal palpation (*fukushin*/腹診), taking the pulse (*myakushin*/脈診), and tongue examination (*zesshin*/舌診) (Eberhard, 2003; Terasawa, 1994; Reißenweber-Hewel, 2014). These methods are compatible and complement our clinical examination.

Kampo medication has been standardized over time, leading to defined herbal drug combinations of a largely standardized dosage (Kuchta, 2014b; Kuchta, 2014c). The aim is to alleviate the leading symptom(s) and restore physical and mental wellbeing, i.e., “balance” (Eberhard, 2003; Terasawa, 1994; Motoo and Yasui, 2024).

While in Western medicine, the term “disease” covers a specific term such as Crohn’s disease or ulcerative colitis, in Kampo medicine, “disease” includes the various symptom patterns specific to the individual patient. For instance, medicines to ameliorate the body constitution (*hozai*/補剤), such as Juzentaihoto (十全大補湯) and Hochuekkito (補中益気湯), are commonly used in cancer patients. Juzentaihoto is employed in cases of fatigue, appetite loss, impaired wound healing, and for its immune-stimulating effects (Cameron et al., 2018). Hence, the use of Juzentaihoto is of great interest alongside chemotherapy (Yamada and Ikuo, 2005). Hochuekkito is used in weak patients having anemia, appetite loss with anorexia, fatigue, slight fever, and night sweat, especially in chronic diseases, including cancer patients, and recovery after infections, operations, or chemotherapy (Cameron et al., 2018; Motoo and Cameron, 2022).

Rikkunshito (六君子湯) is used especially in patients with stomach and digestive problems such as nausea, appetite loss, meteorism, weakness, and cachexia. Thus, it can also be considered as “hozai” for strengthening the body’s constitution, especially in patients with a weak digestive system (Eberhard, 2003; Terasawa, 1994; Reißenweber-Hewel et al., 2023).

Keishikashakuyakuto (桂枝加芍薬湯) is used in Crohn’s disease patients with abdominal distention and cramps (Eberhard, 2003; Terasawa, 1994). Its indication includes patients with a comparatively weak body constitution with abdominal distention, abdominal pain, and impaired digestion, including diarrhea and constipation (Eberhard, 2003; Terasawa, 1994). As Crohn’s disease patients have a weak body constitution, some of the abovementioned remedies might also be suitable.

In Kampo medicine, the symptoms of the various prescriptions might overlap, as can individual herbal ingredients. To find the correct medication, Kampo doctors have a certain image of a symptom complex (*kuketsu*/口訣) in mind when prescribing Kampo medicines (Terasawa, 1994; Hanawa, 1995). As we focus on patients with abdominal complaints and diseases that weaken the body’s constitution, the abovementioned four prescriptions and their respective symptom complexes were chosen for analysis.

With an increasingly individualized therapeutic setting, it might help to apply an individualized (holistic) view of a patients’ body constitution within the Western medicinal context. In traditional Kampo medicine, disease symptoms represent an imbalance in the interplay between the five main organ concepts. This idea is referred to as “five-organ theory (*gozo*/*五臓* five organs)”. As such, the “liver” (*kanzo*/*肝*) is related to metabolic functions, nutrition, and detoxification and is connected to muscle tone. Disturbances can lead to emotional instability with brittle nails and eye fatigue. The “kidneys” (*jinzo*/*腎*) are related to the water household, memory, and concentration, as well as teeth and bone formation and reproduction. Dysbalance can lead—among others—to hearing problems or changes in bone synthesis as well as water distribution. It also correlates to the idea of “inborn energy.” The traditional functions of the “spleen” (*hizo*/*脾*) refer to the maintenance of digestion, blood viscosity, and muscle function. Imbalance can lead to mood swings, appetite loss, and diarrhea. The traditional idea of the “heart” (*shinzo*/*心*) is related to the modern understanding of the organ: blood circulation, sleep–wake rhythm, and consciousness. Its symptoms are inner unrest, tachycardia, hiccup, and also glossitis. The “lung” (*肺*) influences the uptake of oxygen as well as external energy. It is involved in acid–base balance and skin function. In traditional understanding, malfunction leads to symptoms such as dyspnea, fear, and increased sweating. Of course, these “organs” do not stand isolated, but interact with each other (Hanawa, 1995; Otsuka, 1976). In the “five-organ theory” (五臓), each of the five main organ concepts is associated with one of the five classical Eastern elements, namely, the “liver” (肝) with wood (*moku/木*), the “heart” (心) with fire (*hi/火*), the “spleen” (脾) with earth (*do/土*), the “lung” (肺) with metal (*kin/金*), and the “kidney” (腎) with water (*sui/水*).

This ancient theory was streamlined into the theory of *Ki-Ketsu-Sui* (*ki-ketsu-sui ron/気血水論*), i.e., the “three circulating elements of the body”: with Qi (Chinese: 氣, pronounced: “chi,” a concept depicting “vital energy;” Japanese: ki/気), blood (ketsu/血), and water (sui/水), by Yoshimasu Nangai (1749–1813) (Otsuka, 1976). Although it is a typical Japanese concept, Western doctors and patients should be able to intuitively relate, as patients report “lack of energy” (i.e., *Qi*), macro- or microcirculatory problems (i.e., *ketsu*), and water stagnation such as edema (i.e., sui) (Terasawa, 1994; Watanabe, 2019). Subsequently, the term *Qi* was used instead of the Japanese word “*ki*/気” to align with the terminology supported by ICD-11 (WHO, 2019). Accordingly, instead of the Japanese term “water imbalance” or “imbalance of the water household” *(suidoku*/水毒), we employed the international term “fluid disturbance” (Terasawa et al., 1985; WHO, 2019). Even though these descriptions for health conditions are observed worldwide, previous theoretical publications have put forward the idea that in different cultures, people react differently to outer and inner stressors. Therefore, traditional medicines and their diagnostic methods should be employed depending on the patients’ origin (Pandalis and Keil, 2014). In contrast, we hypothesize that due to their descriptive, matter-of-fact nature, Kampo symptom patterns could universally apply.

The purpose of our study was to assess Kampo diagnosis in a Western (German) population. We thus evaluated—for the first time—a traditional Japanese Kampo questionnaire in German patients and volunteers. Although there are internationally available questionnaires for quality of life or clinical scoring in chronic inflammatory bowel diseases, no Western questionnaire addresses all the above-mentioned functions—especially not in the form and structure suitable for standard Kampo anamnesis. We therefore developed a German language questionnaire based on representative Japanese models. As abdominal diagnosis and the treatment of abdominal symptoms remain a main area when treating with herbal medicine, we focused on patients with gastrointestinal diseases. Against common (mis)conceptions, we could show that the reported symptoms were not specific to the underlying disease, pointing out that patients should not be solely visualized within the frame of their disease, but according to the various symptoms they present. These depend on multiple factors such as age, length of disease, body constitution, and mental health, regardless of the patients’ origin (i.e., German or Japanese).

## Materials and methods

### Questionnaire and data acquisition

A traditional Japanese Kampo questionnaire was obtained from the Kitasato University Oriental Medicine Research Center, Tokyo, Japan. It was compared to a Kampo questionnaire from Keio University, Department of Kampo Medicine (Tokyo) and to a questionnaire of Ehime Prefectural Hospital (Ehime, Matsuyama). The original Japanese questionnaire was translated into German (KK), adapted for German patients (SC), and re-translated into Japanese and English (KK, SC, and DY) (Supplementary Material 1). It was approved by DY and KW. It consists of 20 sections, including 108 questions on general condition, mental health, sleep, pain sensation, skin, complaints of the head, eyes and vision, nose, mouth, ears and hearing, neck and swallowing, voice, chest, abdomen, appetite, nutrition, stool properties, urination, joint flexibility, heat sensation, menstruation, and a question on the helpfulness of the questionnaire itself. The patients were given the following answer choices: no complaints, seldom, occasional, and often.

Ethical approval was obtained from the Ethics Committee of the University Medicine Göttingen (40/3/21). All included patients gave written informed consent to participate after oral and written explanation of the study. The inclusion criteria were age above 20 and the ability and willingness to give informed consent, as well as a stable general condition. The exclusion criteria were lack of informed consent and lack of ability to fill out the questionnaire, as well as poor general health condition.

Patients visiting the outpatient clinic of the Department of Gastroenterology, Hann. Münden and the University Medical Center Göttingen (UMG) were asked whether they would like to participate and fill out the questionnaire. Those willing and able to give informed consent were then recruited into the study. For comparison, 20 Japanese patients with abdominal symptoms were recruited in a Japanese general hospital (Ehime Prefectural Central Hospital, Matsuyama, Japan), accordingly. As all participants were outpatients and were able to fill out the questionnaire, they presented in a stable general condition. The control group consisted of 20 German volunteers without gastrointestinal disease.

Five different groups of patients were analyzed: patients with gastrointestinal cancers (tumor group), ulcerative colitis (UC), Crohn’s disease, irritable bowel syndrome (IBS), and gastrointestinal disease, other than the abovementioned diseases, i.e., dyspepsia). As the study consists of real-world data, no stratification was done for the therapeutical setting. Cancer patients were included after primary tumor resection, and various therapeutical settings were allowed. However, most cancer patients attended follow-up.

### Statistical analysis

The individual answers were classified using a numerical system (with no complaint being 0, seldom = 1, occasional = 2, and often = 3 points). Symptom scores for the individual symptom blocks (i.e., mental health, and sleep) were obtained by adding the individual points per block followed by division with the number of questions. For five different groups of patients as well as controls, box plots were generated. Significant differences between two groups were evaluated using the Mann–Whitney U test. The significance level was estimated as *p < 0.05 and ** for p < 0.01.

Significant differences within single questions were evaluated by the Kruskal–Wallis test. A paired comparison between the different entities was done by the Dwass–Steel–Critchlow–Fligner test.

In the second step, the answers were transformed into the following numerical values for cluster analysis: with no complaint being 1, seldom = 2, occasional = 3, and often = 4 points. Missing values remained empty. Using the MetaboAnalyst Software (Version 5.0), the program algorithm replaced these missing values with one-fifth of the smallest numerical value. Subsequently, a principal component analysis was done using a matrix depicting the five most weighted components. The two main components were depicted in two-dimensional plots, with a 95% confidence interval.

The results of multivariate cluster analysis are displayed as heatmaps. The presetting was chosen following the cluster method by Ward, with scaling and coloring by software-inherent algorithms. To answer whether symptom constellations are depicted by individual patients/diseases, clusters were identified, and individual cases were counted by hand. Statistical differences were analyzed using the Fisher’s exact test.

For further analysis, defined symptom combinations for Kampo medicines (*Kuketsu*), such as *Juzentaihoto*, *Hochuekkito*, *Rikkunshito*, and *Keishikashakuyakuto,* were written into a fourfold table with the symptom constellation estimated “correct” and the presence or absence of a particular diagnosis (i.e., Crohn’s disease yes/no). The Fisher’s exact test was used to verify whether a defined symptom group was significantly overlapping with one of the patients’ groups.

In order to analyze whether traditional Kampo prescriptions were covering individual diseases or rather individual complaints, heatmaps were generated, taking into account not only the individual symptoms but also traditional classifications following the concept of energy/Qi (*ki*/気), blood (*ketsu*/血), and water (*sui*/水), as well as the older five-organ theory (i.e., spleen) or the five elements (i.e., water; these latter are not shown). To this end, symptoms were re-classified according to these traditional concepts (DY and NS).

## Results

### Patient collective

**FIGURE 1:**
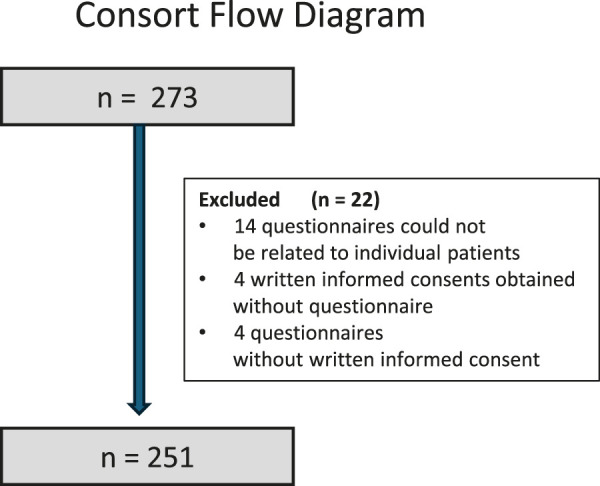
Consort flow diagram.

In total, we recruited 251 participants. Figure 1 shows the CONSORT flow diagram for these patients. Individual patient groups are presented in Table 1. The group of Japanese patients had a mean age of 52.8 (±23.5) years. The controls were slightly younger, with a mean age of 47.4 (±11.5) years. The duration of the disease varied from 1 month (a cancer patient) to 540 months (a patient with Crohn’s disease). The average disease duration was 211 months. As cancers were mostly removed, this group has been termed “tumor patients.”

**TABLE 1:** Patient background.

Individual patient groups	Number of patients (%)	Age (years) mean ± SD	Sex male/female
	Total: 251	All: 56.0 ± 17.9 years Male: 57.2 ± 16.9 years Female: 55.2 ± 18.6 years	107 (42.6%)/144 (57.4%)
Tumor patients	58 (23.1%)	65.5 ± 12.0	29/29
Ulcerative colitis (UC)	18 (7.2%)	48.4 ± 16.7	7/11
Crohn’s disease (CD)	35 (13.9%)	48.8 ± 16.8	13/22
Irritable bowel syndrome (IBS)	17 (6.8%)	53.6 ± 24.0	4/13
Other GI diseases	103 (41.0%)	56.4 ± 18.4	43/60
Controls	20 (8.0%)	47.4 ± 11.5	11/9

### Symptom scores—mental, general health, and sleep quality

Questions describing mental health included the feeling of depression, anxiety, forgetfulness, fatigue, irritability, being easily frightened, and mood swings.

**FIGURE 2:**
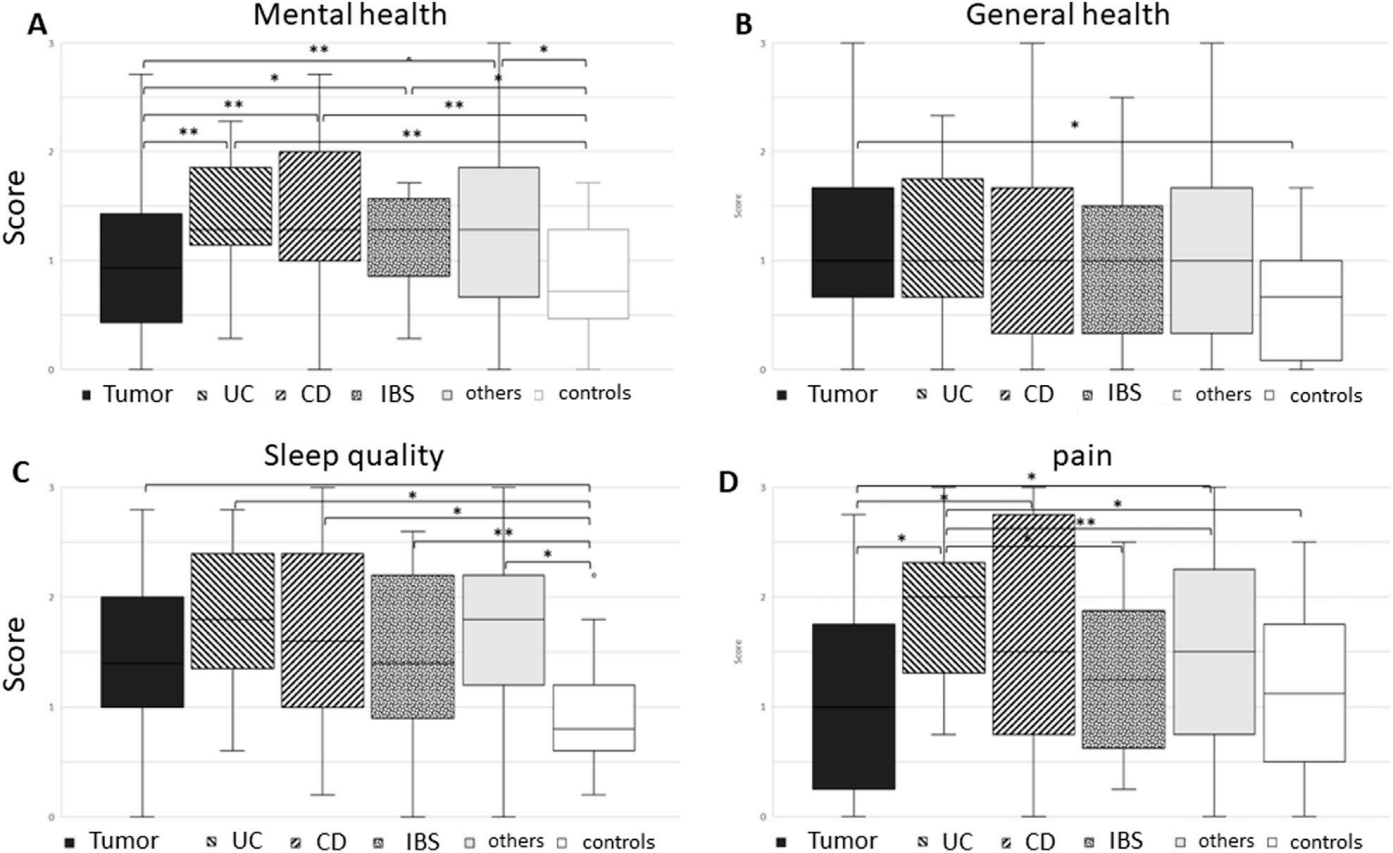
Boxplot of various groups: tumor, ulcerative colitis (UC), Crohn’s disease (CD), irritable bowel syndrome (IBS), others, and controls in view of questions regarding **(A)** mental health, **(B)** general health, **(C)** sleep quality, and **(D)** pain. Although tumor and control groups complained about significantly less mental symptoms when compared to inflammatory and irritable bowel disease patients, the tumor patients in our study reported significantly more general health symptoms compared to controls. Sleep quality was significantly better in controls compared to all patient groups. Pain was mostly reported in patients with inflammatory bowel disease with significant differences especially in UC patients (Mann–Whitney U test) between two groups are given in brackets with *p < 0.01 and **p < 0.05.

Patients with inflammatory bowel disease (IBD) and irritable bowel syndrome (IBS) showed significantly more symptoms in comparison to the tumor and control groups (Figure 2A).

The questions on general health included frequent or decreased sweating, paresthesia in hands and feet (such as tingling or numbness), and vertigo or the feeling of sudden darkness (when standing up).

Although control patients reported fewer of these symptoms than all the other groups, the difference only became significant in comparison to *tumor* patients, with a rather high standard deviation (Figure 2B).

Sleep quality included questions about inner unrest, problems falling asleep or sleeping, frequent dreams, or daytime sleepiness. *All patient groups* reported significant sleeping problems (Figure 2C). The most prominent symptoms were inner unrest and problems falling asleep or sleeping through (Kruskal–Wallis test).

The pain questionnaire included questions about joint pain (small joints such as fingers and large joints such as knees and hip) and back and muscle pain.

Patients with *Crohn’s disease* reported the highest pain score with high standard deviation. *Ulcerative colitis* patients were closest with a significantly higher symptom score compared to the other groups (Figure 2D), with muscle pain being less relevant.

### Symptom scores—sensory organs

**FIGURE 3:**
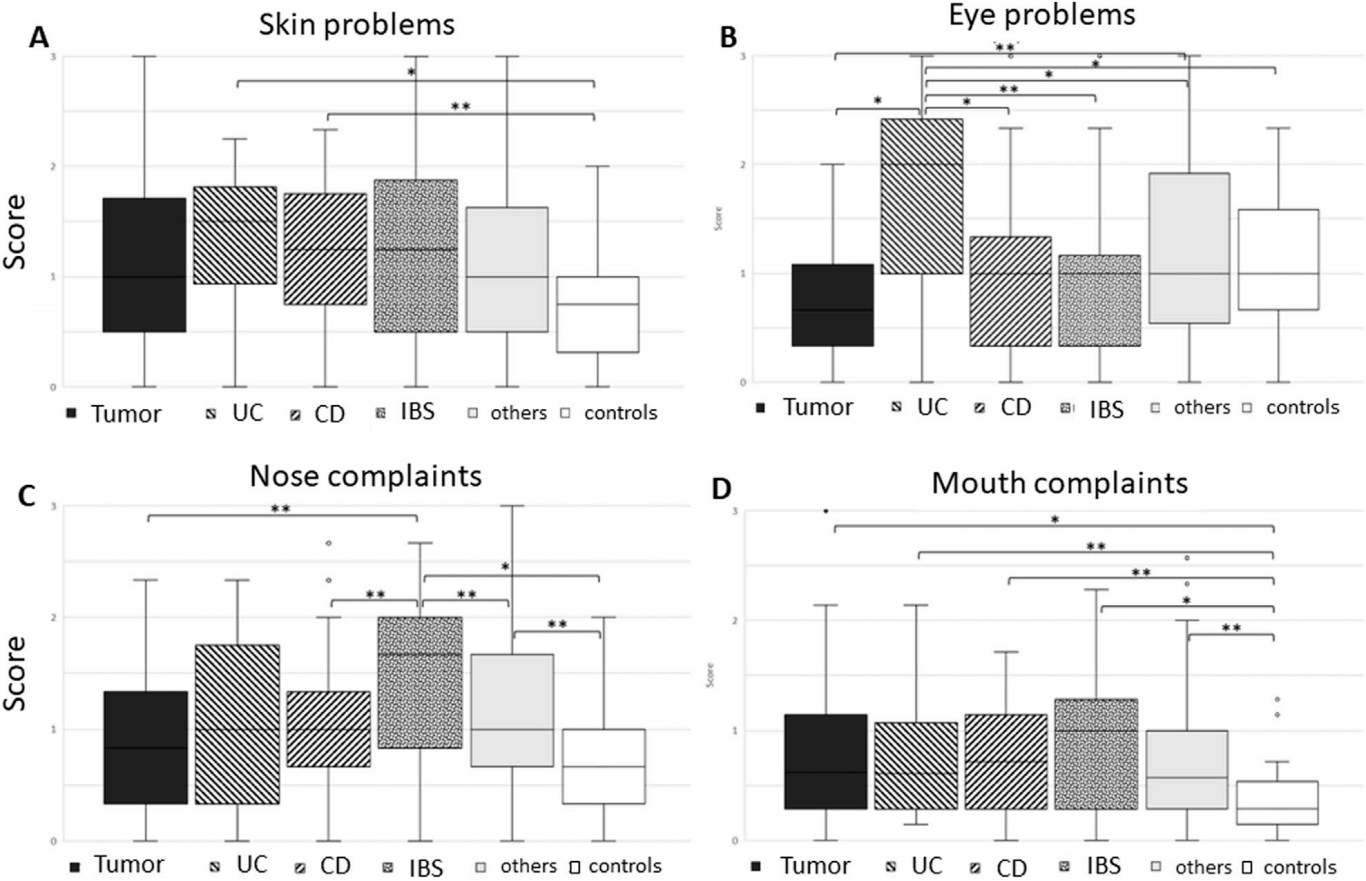
Boxplot of various groups: tumor, ulcerative colitis (UC), Crohn’s disease (CD), irritable bowel syndrome (IBS), others, and controls in view of questions regarding **(A)** skin problems, **(B)** eye problems, **(C)** nasal complaints, and **(D)** mouth complaints. Skin problems were mostly reported in inflammatory bowel disease patients, with eye problems being most prominent in CU patients, while nasal complaints were most reported in IBD patients. Mouth problems were slight, however present in all patient groups when compared to controls (Mann–Whitney U test). Significant differences between two groups are given in brackets, with *p < 0.01 and **p < 0.05.

The questionnaire further included questions on sensory organs such as the skin, eyes, nose, and mouth. As for skin problems, questions were asked about dry and moist skin, itchiness, brittle nails, and hair loss. Here, patients with *inflammatory bowel disease* reported most problems (Figure 3A).

Eye problems included questions on tired/strained eyes, red eyes, and rings underneath the eyes. Here, patients with *ulcerative colitis* reported significantly more symptoms, also in comparison with Crohn’s disease patients (Figure 3B). The symptoms of red eyes and rings underneath the eyes were the most prominent (Kruskal–Wallis test).

Nose complaints included frequent sneezing, rhinorrhea, mucus in the throat, and problems breathing through the nose or blocked nose (Figure 3C). Here, *IBS patients* reported most symptoms, with sneezing being the most prominent (*p* = 0.02), Kruskal–Wallis test).

Mouth complaints included dry mouth, bitter taste, salivation, changed taste, painful tongue, frequent mouth inflammation, and dry lips. All patients reported more symptoms than the control group, with *IBS patients* showing the highest differences, however, with a high standard deviation (Figure 3D).

### Symptom scores—abdomen, appetite, fecal habits, and cold sensation

Questions regarding the abdomen were abundant, even though the score was not designed for gastrointestinal patients. They included enquiries about belching, nausea, vomiting, abdominal pain, distended abdomen/meteorism, abdominal noises, and problems with digestion.

**FIGURE 4:**
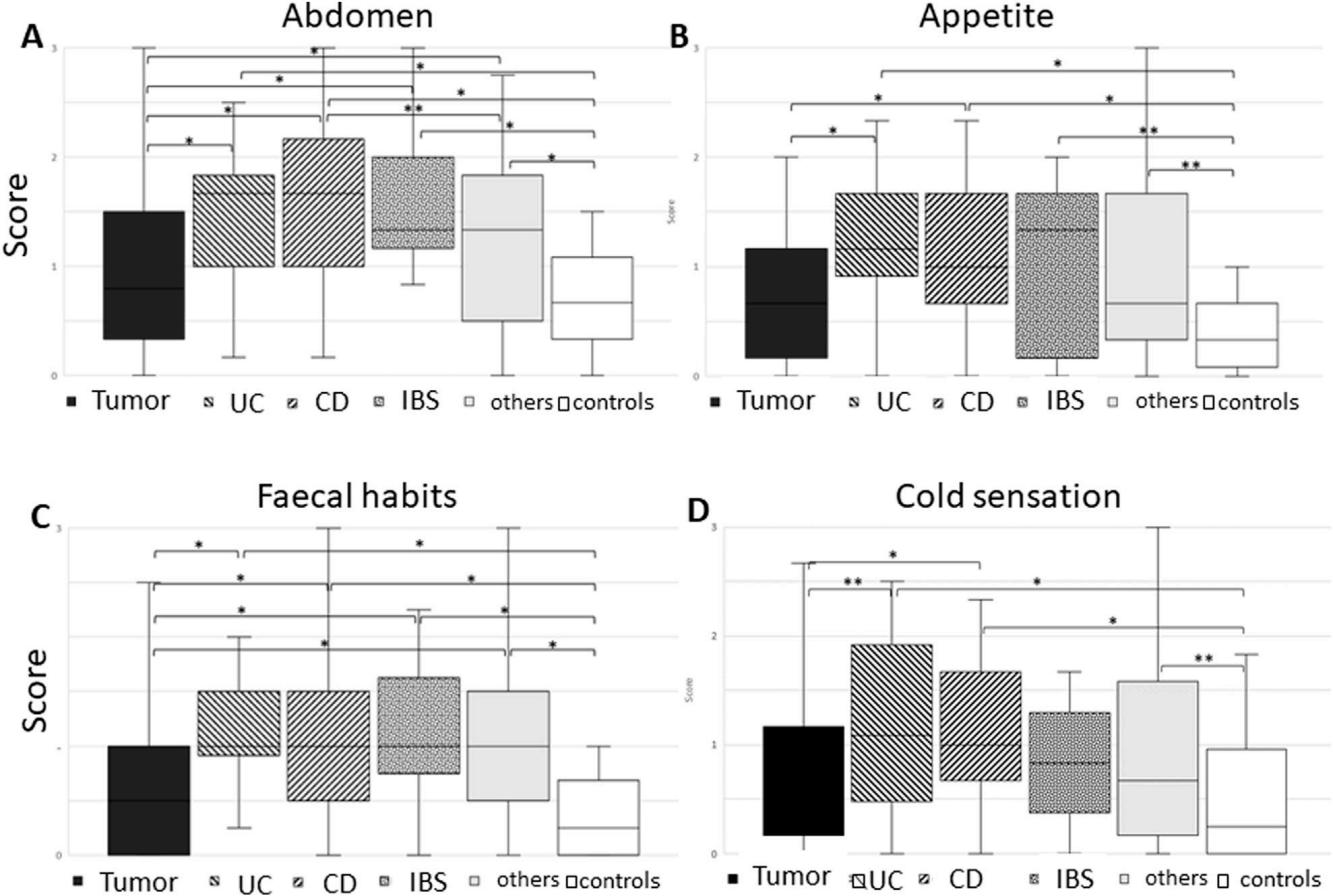
Boxplot of the various groups: tumor, ulcerative colitis (UC), Crohn’s disease (CD), irritable bowel syndrome (IBS), others, and controls in view of questions regarding **(A)** abdominal symptoms, **(B)** changes in appetite, **(C)** fecal habits, and **(D)** cold sensation. Note that all controls had less symptoms compared to the patient groups, with our tumor patients reporting less abdominal symptoms, changes in appetite, and fecal habits. Tumor and IBD patients reported less cold sensation, while chronic inflammatory bowel disease patients showed the highest score (Mann–Whitney U test). Significant differences between two groups are given in brackets, with *p < 0.01 and **p < 0.05.

Overall, *controls and tumor patients*—the latter mostly after primary cancer resection—reported significantly *less symptoms* compared to patients with inflammatory or irritable bowel disease as well as patients with other abdominal complaints (Figure 4A). Most prominent symptoms in this complex were vomiting, meteorism, and digestive complaints (Kruskal–Wallis test).

Questions on appetite included loss or increased appetite as well as missing the joy of eating. Controls reported significantly less symptoms than all patient groups. In comparison, tumor patients did comparatively well (Figure 4B).

Similarly, fecal habits were changed in comparison to controls. Questions included constipation, diarrhea, and stool irregularities with alternation of constipation and diarrhea as well as hemorrhoids. Only the reported fecal habits in tumor patients showed no significant change toward controls (Figure 4C).

Cold sensation as a sign of physical draining or “*yin* status” was examined for hands, feet, bottom, belly, back, and general cold sensation.

Similar to the other groups, also for cold sensation, controls were doing best, with no significant difference between tumor and IBD patients, while inflammatory bowel disease patients showed the highest score, followed by “others” (Figure 4D).

### Heatmap correlating all answers with individual patients

**FIGURE 5:**
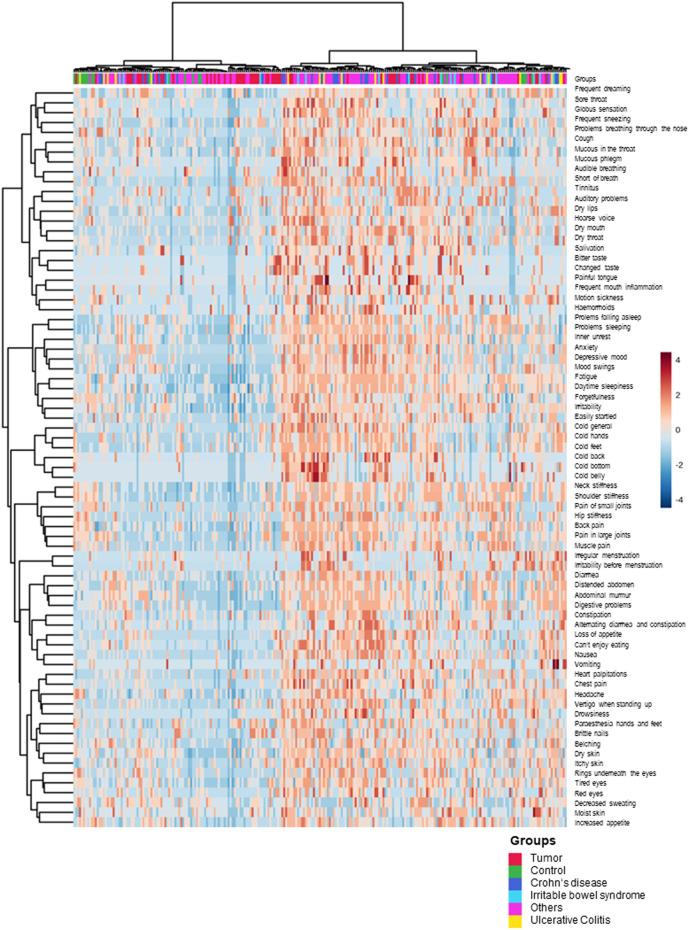
Heatmap of the various groups: tumor, UC, CD, IBS, and others in view of symptom expression. The upper line shows the distribution of the various groups. No clustering of patients/controls and symptom score was observed (MetaboAnalyst 5.0).

To depict whether the various patient groups and controls cluster with the described symptom score (i.e., “not at all,” “rarely,” “occasional,” and “common”), a heatmap was constructed, where *red* stands for applicable and *blue* for not applicable. Every column represents one patient, and every line depicts a question. Correlations between individual patients are marked by the tree diagram at the top (Figure 5). Please note that the individual patients are intercalated, regardless of the underlying disease. For better visualization, the same figure has been regrouped according to the disease groups and added as Supplementary Figure 1. As there is no clustering between the symptom score and patient group (patients are scattered), we can conclude that specific symptoms do not accumulate within a specific group of patients; instead, the expression of symptoms is rather individual.

### Heatmap correlating Japanese and German patients

**FIGURE 6:**
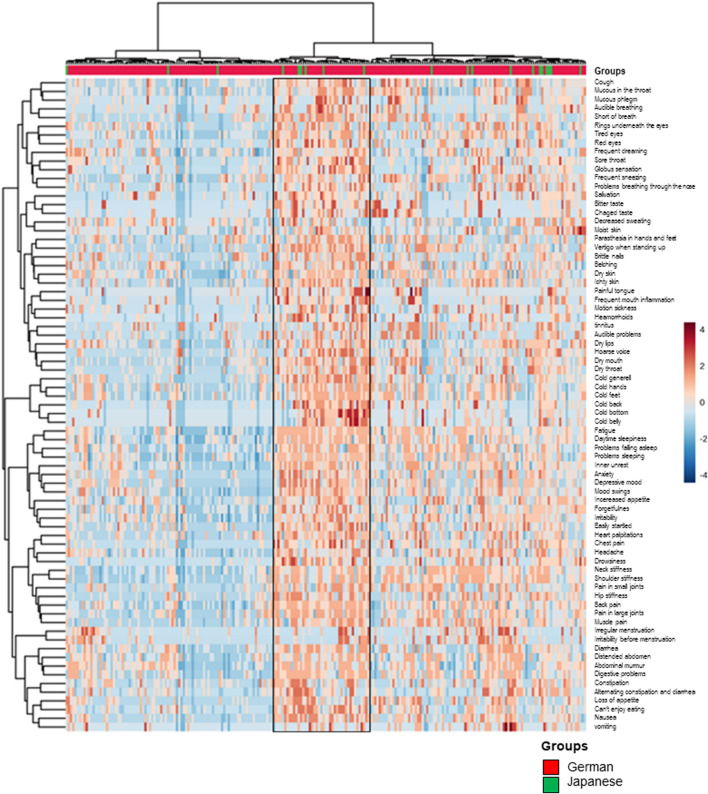
Heatmap—comparison of answer profiles from Japanese and German patients. Symptom expression did not cluster with either group (MetaboAnalyst 5.0).

The genetic background varies according to the country of origin, and also the perception of symptoms might vary according to the cultural background. Hence, it has been proposed that, especially in traditional medicines, traditional diagnostic and therapeutic methods might not apply to groups not familiar with the traditional medical system (Pandalis and Keil, 2014). We thus correlated answers from Japanese and German patients depicted within a heatmap (Figure 6). German and Japanese patients were intercalated. Although a small group of individual patients—regardless of their origin—shared individual symptoms, there was no separation of German and Japanese patients. Whilst the computer analysis depicted a field with the clustering of symptomatic answers, the two-sided Fisher’s exact test did not show a significant difference in German and Japanese patients within the field, i.e., patients with a high symptom score.

### Heatmap correlating symptoms for Juzentaihoto use with individual patients

In traditional medical systems, such as Japanese Kampo medicine, symptom constellations (so-called *kuketsu*) have been described for each traditional herbal combination prescription. The composition of herbal drug combinations has been clearly defined, standardized, and is controlled according to good manufacturing (GMP) and good laboratory practice (GLP) guidelines in Japan. Nevertheless, doctors with specialization in Kampo medicine can adapt and modify the medication, if used in the form of decoction.

In the following section, we analyzed the questionnaire of our German patients for various questions of certain well-described Kampo prescriptions, which would be administered in the setting of tumor patients, inflammatory bowel disease patients, IBS patients, or other patients with other gastrointestinal disorders.

*Juzentaihoto* (十全大補湯*)* is a combination of 10 herbal drugs: *Panax ginseng* root (2–3 g), *Atractylodes lancea* rhizome (3–4 g), *Poria cocos* sclerotium (3–4 g), *Glycyrrhiza uralensis* rhizome (1.5–2 g), *Angelica acutiloba* root (3–4 g), *Paeonia lactiflora* root (3 g), *Cnidium officinale* rhizome (3 g), *Rehmannia glutinosa* root (3–4 g), *Astragalus membranaceus* root (3 g), and *Cinnamomum cassia* bark (3 g) (Eberhard, 2003; Terasawa, 1994).

Like *Hochuekkito (*補中益気湯, *see below)*, *Juzentaihoto* is used as a so-called *hozai* to ameliorate a weakened body constitution (equivalent to adaptogens in the West) and especially in tumor patients for recovery after operation or chemotherapy (Figure 6a) (Cameron et al., 2018; Yamada and Ikuo, 2005; Motoo and Cameron, 2022).

**FIGURE 7:**
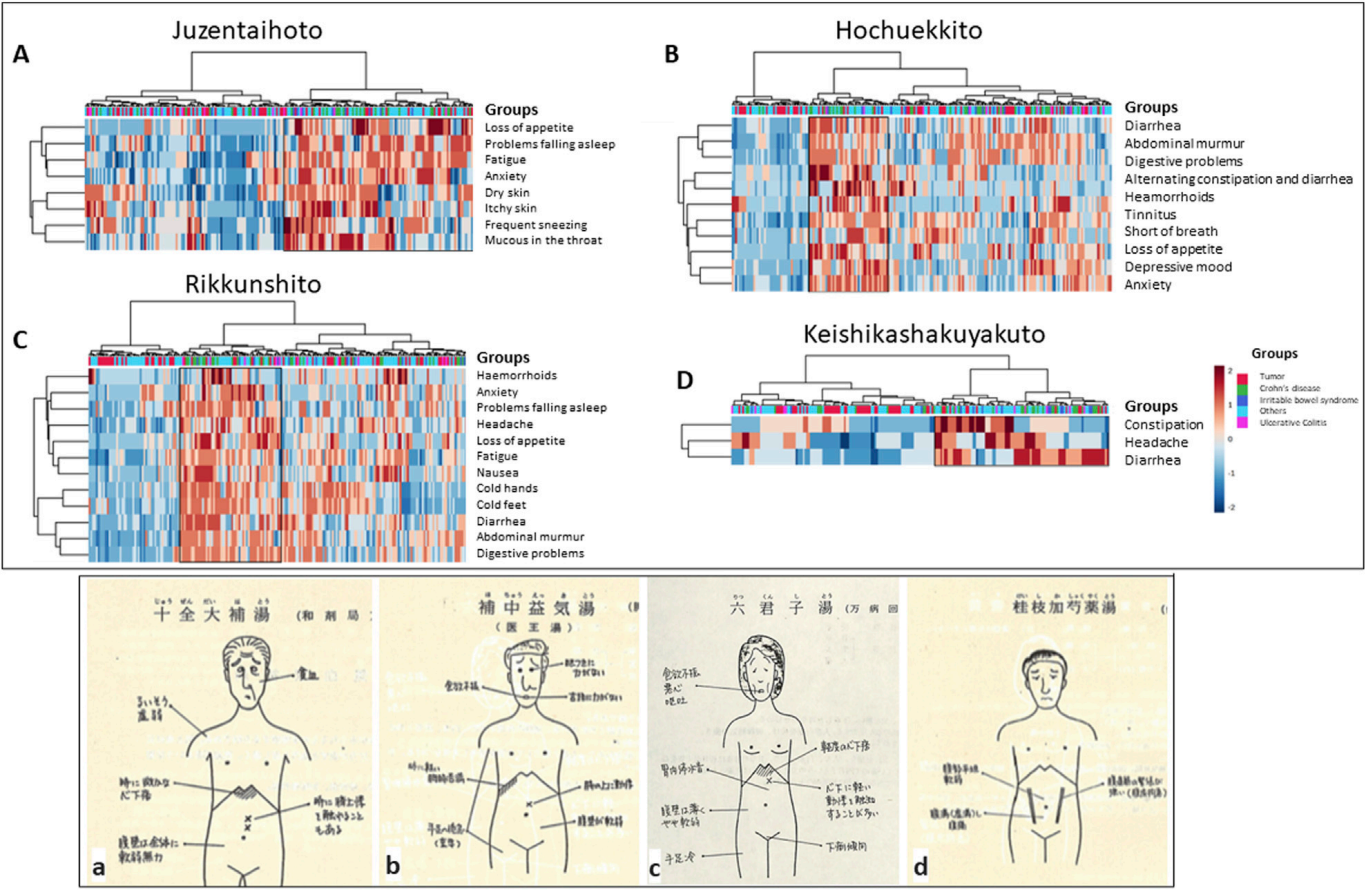
Heatmaps depicting Kampo-specific symptoms in various groups of German patients and controls (MetaboAnalyst 5.0). Patients with typical symptoms are marked within the box. **(A)** *Juzentaihoto*: the best fitting had tumor patients and patients with Crohn’s disease (two-sided Fisher’s exact test). **(B)** *Hochuekkito*: the best fitting had tumor patients (two-sided Fisher’s exact test). **(C)** *Rikkunshito*: the best fitting had patients with Crohn’s disease (two-sided Fisher’s exact test). **(D)** *Keishikashakuyakuto*: the best fitting had patients with tumors and Crohn’s disease (two-sided Fisher’s exact test). **(a–d)** depict modern cartoons of various abdominal signs for different preparations: **(a)** sketch of signs for Juzentaihoto (十全大補湯), **(b)** Hochuekkito (補中益気湯), **(c)** Keishikashakuyakuto (桂枝加芍薬湯), and **(d)** Rikkunshito (六君子湯) (Takayama, 1991), Courtesy of DY).

In our analysis, we could extract one group of patients (marked in the box at the right of the heatmap) who showed symptoms typical for the prescription *Juzentaihoto*. It included 104 patients, of which 20.2% (n = 21) were tumor patients, 3.8% (n = 4) were ulcerative colitis patients, 22.1% (n = 23) patients with Crohn’s disease, 7.7% (n = 8) were IBD patients, and 46.2% (n = 48) were patients with other gastrointestinal complaints (Figure 7A). Table 2 shows the percentages of patients who would suit *Juzentaihoto* treatment within the various groups. Note that patients with Crohn’s disease suited the most (*p* = 0.02), while patients with tumors suited the least (*p* = 0.024) when compared to the remaining study collective. We can conclude that symptoms do not depict a specific disease but can account for the choice of treatment, also in German patients.

**TABLE 2:** Symptom patterns within various groups suiting the prescriptions Juzentaihoto, Hochuekkito, Rikkunshito, and Keishikashakuyakuto.

			Juzentaihoto		Hochuekkito		Rikkunshito		Keishika shakuyaku to	
Patient	all	n	%	p	%	p	%	p	%	p
Tumor	58	21	36.2	0.02 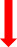	8.6	0.007 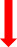	5.5	n.s.	22.4	0.001 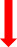
Ulcerative colitis	12	4	33.3	n.s.	16.7	n.s.	41.7	n.s.	41.7	n.s.
Crohn‘s disease	34	23	67.6	0.024 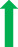	32.4	n.s.	47.1	0.005 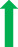	67.6	0.008 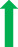
IBS	13	8	61.5	n.s.	23.1	n.s.	30.8	n.s.	76.9	0.04 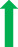
GI-disorders	94	48	51.1	n.s.	24.4	n.s.	22.3	n.s.	46.8	n.s.

### Heatmap correlating symptoms for Hochuekkito use with individual patients

*Hochuekkito* (補中益気湯) contains *Astragalus membranaceus* radix (4 g), *Atractylodis lanceae* rhizome (4 g), *Panax ginseng* root (4 g), *Angelica acutiloba* root (3 g), *Citrus reticulata* pericarp (2 g), *Ziziphus jujuba* fruit (2 g), *Bupleurum falcatum* root (2 g), *Glycyrrhiza uralensis* rhizome (1–1.5 g), *Cimicifuga simplex* rhizome (1 g), and *Zingiber officinale* rhizome (0.5–1 g) (Eberhard, 2003; Terasawa, 1994).

*Hochuekkito* is used to ameliorate the body constitution in weak patients and support recovery after tumor treatment or infections (Figure 7B) (Cameron et al., 2018; Motoo and Cameron, 2022).

With the symptom combination that would be suitable for *Hochuekkito*, our analysis showed a defined group (marked within a black box) (Figure 7B). Patients depicted within the box included 44 patients suitable for the prescription *Hochuekkito*. They consist 11.4% (n = 5) tumor patients, 4.5% (n = 2) patients with ulcerative colitis, 25% (n = 11) patients with Crohn’s disease, 6.8% (n = 3) IBS patients, and 52.3% (n = 23) patients with varying gastrointestinal problems. Table 2 shows the percentages of patients who would suit *Hochuekkito* treatment. Although in all patient groups a few patients suited the characteristics for the prescription, our tumor patients suited significantly less, i.e., they had a significantly better symptom profile.

### Heatmap correlating symptoms for Rikkunshito use with individual patients

*Rikkunshito* (六君子湯) is used to strengthen the body’s constitution, especially in patients with weak digestion (Figure 6c) (Cameron et al., 2018; Motoo and Cameron, 2022; Reißenweber-Hewel et al., 2023). It contains *Panax ginseng* root (4 g), *Atractylodis lanceae* rhizome (4 g), *Poria cocos* sclerotium (4 g), *Pinellia ternata* tuber (4 g), *Citrus reticulata* pericarp (2 g), *Ziziphus jujuba* fruit (3 g), *Glycyrrhiza uralensis* rhizome (2 g), and *Zingiber officinale* rhizome (0.5 g) (Eberhard, 2003; Terasawa, 1994).

Patients best fitting to the respective questions are marked within the black box depicted in the heatmap (Figure 7C): 55 patients. This group consists of16.4% (n = 9) tumor patients, 9.1% (n = 5) patients with ulcerative colitis, 29.1% (n = 16) patients with Crohn’s disease, 7.3% (n = 4) patients with IBD, and 38.2% (n = 21) patients with other gastrointestinal problems. Table 2 also shows the percentages of patients who would suit *Rikkunshito* treatment within the various groups. Although all patient groups had features suiting the prescription *Rikkunshito*, again patients with Crohn’s disease showed a significant association with the symptom combination defined by the prescription, compared to the remaining study collective (*p* = 0.005).

### Heatmap correlating symptoms for Keishikashakuyakuto use with individual patients

*Keishikashakuyakuto* (桂枝加芍薬湯) contains *Paeonia lactiflora* root (6 g), *Cinnamomum cassia* bark (4 g), *Ziziphus jujuba* fruit (4 g), *Glycyrrhiza uralensis* rhizome (2 g), and *Zingiber officinale* rhizome (1 g). It is used in patients with a weak body constitution, abdominal cramps, and irregular stools (Figure 7D) (Eberhard, 2003; Terasawa, 1994).

The heatmap depicts 97 patients (marked within a black box at the right), which is some of these symptoms from our questionnaire (Figure 7D). This group consists of 13.4% (n = 13) tumor patients, 9.4% (n = 5) patients with ulcerative colitis, 23.7% (n = 23) patients with Crohn’s disease, 10.3% (n = 10) patients with IBS, and 45.4% (n = 44) patients with other gastrointestinal problems. Table 2 again shows the percentages of patients who would suit *Keishikashakuyakuto* treatment within the various groups. In our study collective, Crohn’s disease patients suit this medication best, followed by IBS-patients. Again, our tumor patients had significantly less symptoms suiting the prescription when compared to the remaining study collective (*p* < 0.001).

### Balance of energy, blood, and water household (Ki-Ketsu-Sui)

#### Energy household

Specific symptoms point to an imbalance of energy, blood/circulation, or fluid disturbance (Terasawa, 1994; Otsuka, 1976; Watanabe, 2019).

Symptoms of Qi-excess are hypertension, overweight, or a red face, while symptoms of Qi-deficiency are lack of energy, fatigue, sleeping disorder, cold sensation, inner-heat and outer cold or frequent sweating, shortness of breath, heart palpitations, loss of appetite, frequent urination, or depressive mood.

Qi-stagnation is characterized by mood swings, irritability, depression, and a distended abdomen. Some patients express problems sleeping or falling asleep, inner unrest, headache, and motion sickness. Others express belching, nausea, vomiting, constipation, or intestinal congestion as well as palpitations.

**FIGURE 8:**
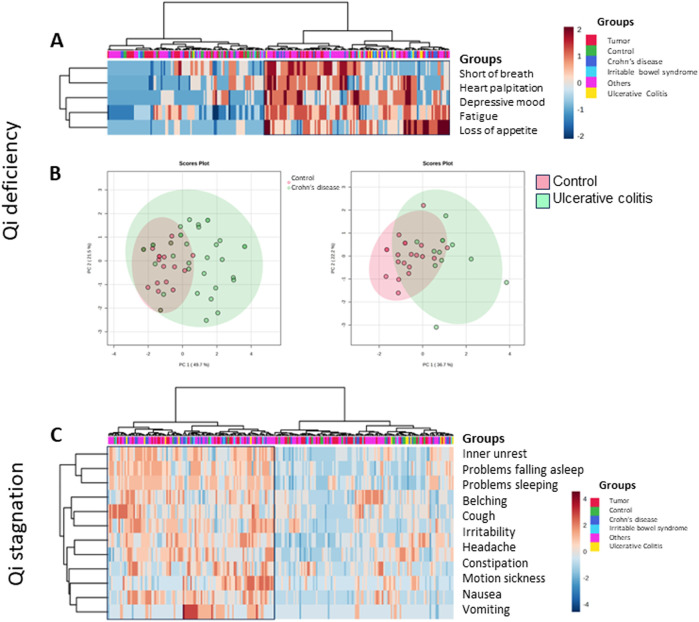
Heatmap depicting Kampo-specific symptoms for the lack of energy in the various groups of German patients and controls (MetaboAnalyst 5.0). Patients with typical symptoms are marked within the box. **(A)** Qi-deficiency was predominant in inflammatory bowel disease patients (two-sided Fisher’s exact test). **(B)** Primary component analysis for Qi-deficiency. The plot depicts two questions with the maximal difference of controls (red) compared to patients with Crohn’s disease (left, green) or ulcerative colitis (right, green), within a 95% confidence interval. **(C)** Heatmap depicting Kampo-specific symptoms for Qi-stagnation in the various groups of German patients and controls (MetaboAnalyst 5.0). Patients with typical symptoms are marked within the box. Although 111 patients showed symptoms of Qi-stagnation, in none of the patient groups it became significant (two-sided Fisher’s exact test).

Approximately half of our patients had symptoms of Qi-deficiency (marked by the box on the right, Figure 8A), 20% (n = 25) of those were tumor patients, 8.8% (n = 11) had ulcerative colitis, 19.2% (n = 24) had Crohn’s disease, 6.4% (n = 8) had IBS, 44% (n = 55) had other gastrointestinal complaints, and 1.6% (n = 2) were controls. Table 3 shows the percentages of patients with Qi-deficiency, Qi-stagnation, blood deficiency, and fluid disturbance within the various groups. Qi-deficiency was not predominant in our tumor patients, however, it was significantly different in patients with inflammatory bowel disease, with *p* = 0.007 compared to the remaining study collective for UC patients and *p* = 0.041 for Crohn’s disease patients. For control participants, symptoms of Qi-deficiency were significantly less when compared to the remaining study population with *p* < 0.001. Primary component analysis showed the difference in controls and patients with inflammatory bowel disease for the symptoms of Qi-deficiency in a scatter plot. Controls form a much more homogeneous core, while especially patients with Crohn’s disease, but also ulcerative colitis patients show scattered symptoms (Figure 8B).

**TABLE 3:** Symptoms of Qi-deficiency, Qi-stagnation, and “blood”deficiency within various groups.

			Qi-deficiency		Qi-stagnation		Blood deficiency		Water imbalance	
Patient	all	n	%	p	%	p	%	p	%	p
Tumour	58	25	43.1	n.s.	37.9	n.s.	63.8	n.s.	36.2	n.s.
Ulcerative colitis	12	11	91.7	0.07 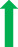	41.7	n.s.	58.3	n.s.	8.3	n.s.
Crohn‘s disease	34	24	70.6	0.04 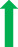	58.8	n.s.	70.6	n.s.	26.5	n.s.
IBS	13	8	61.5	n.s.	61.5	n.s.	69.2	n.s.	46.2	n.s.
GI-disorders	94	55	58.5	n.s.	54.3	n.s.	64.9	n.s.	31.9	n.s.
Controls	20	2	10.0	<0.001 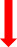	25.0	0.036 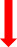	45.0	n.s.	25	n.s.

In our patient collective, a group of 111 patients presented with Qi-stagnation (marked with the box on the left, Figure 8C). This symptomatic group consists of 19.8% (n = 22) tumor patients, 4.5% (n = 5) patients with ulcerative colitis, 18% (n = 20) patients with Crohn’s disease, 7.2% (n = 8) patients with IBD, 45.9% (n = 51) patients with other gastrointestinal complaints, and 4.5% (n = 5) controls. None of the patient groups showed significant clustering for symptoms regarding Qi-stagnation; however, the control group was situated at the lower end, which was significant in comparison to the remaining study collective (*p* = 0.036) (Table 3).

#### Blood circulation (ketsu)

Deficiency of blood circulation (*Oketsu*/瘀血) corresponds to symptoms such as brittle nails, hair loss (i.e., during combing), headache, tired eyes, red eyes, rings underneath the eyes, painful tongue, menstruation disorders, early menopause, constipation/congestion, hemorrhoids, and cold sensation at the extremities (hands/feet), back, bottom, belly, or general feeling of cold.

**FIGURE 9:**
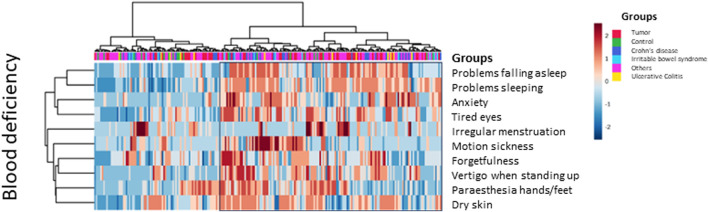
Heatmap–blood deficiency. Questions depicting Kampo-specific symptoms for blood deficiency in various groups of German patients and controls (MetaboAnalyst 5.0). Patients with typical symptoms are marked within the box. Although 147 patients showed symptoms of blood deficiency, more than 50% in every patient group, in none of the groups an association of diagnosis and blood deficiency became significant (two-sided Fisher’s exact test).

A total of 147 patients presented with symptoms of blood deficiency (depicted in the right side of the heatmap, Figure 9). This symptomatic group consisted of 25.2% (n = 37) tumor patients, 4.8% (n = 7) patients with ulcerative colitis, 16.3% (n = 24) patients with Crohn’s disease, 6.1% (n = 9) patients with IBS, 41.5% (n = 61) patients with other gastrointestinal complaints, and 6.1% (n = 9) controls. Table 3 shows the percentages of patients with blood deficiency within the various groups. Although more than 50% within each group showed signs of blood deficiency, none of the patients showed significant differences regarding blood deficiency, when compared to the remaining study population.

#### Water household (*sui*) and imbalance (*suidoku*/水毒)

Symptoms of fluid disturbance in our questionnaire cover frequent or decreased sweating, fluid retention, vertigo when standing up, dry skin, motion sickness, rhinorrhea, mucus in the throat and dry mouth or throat, salivation, mucus phlegm, cough, constipation/congestion or diarrhea or alteration in stool habits, as well as polyuria or oliguria, less frequent urination, or frequent urination as well as frequent nycturia.

Some of the symptoms overlap with imbalance of blood circulation or energy deficiency; however, they have to be seen as bouquet, shown as clusters in the main component analysis.

**FIGURE 10:**
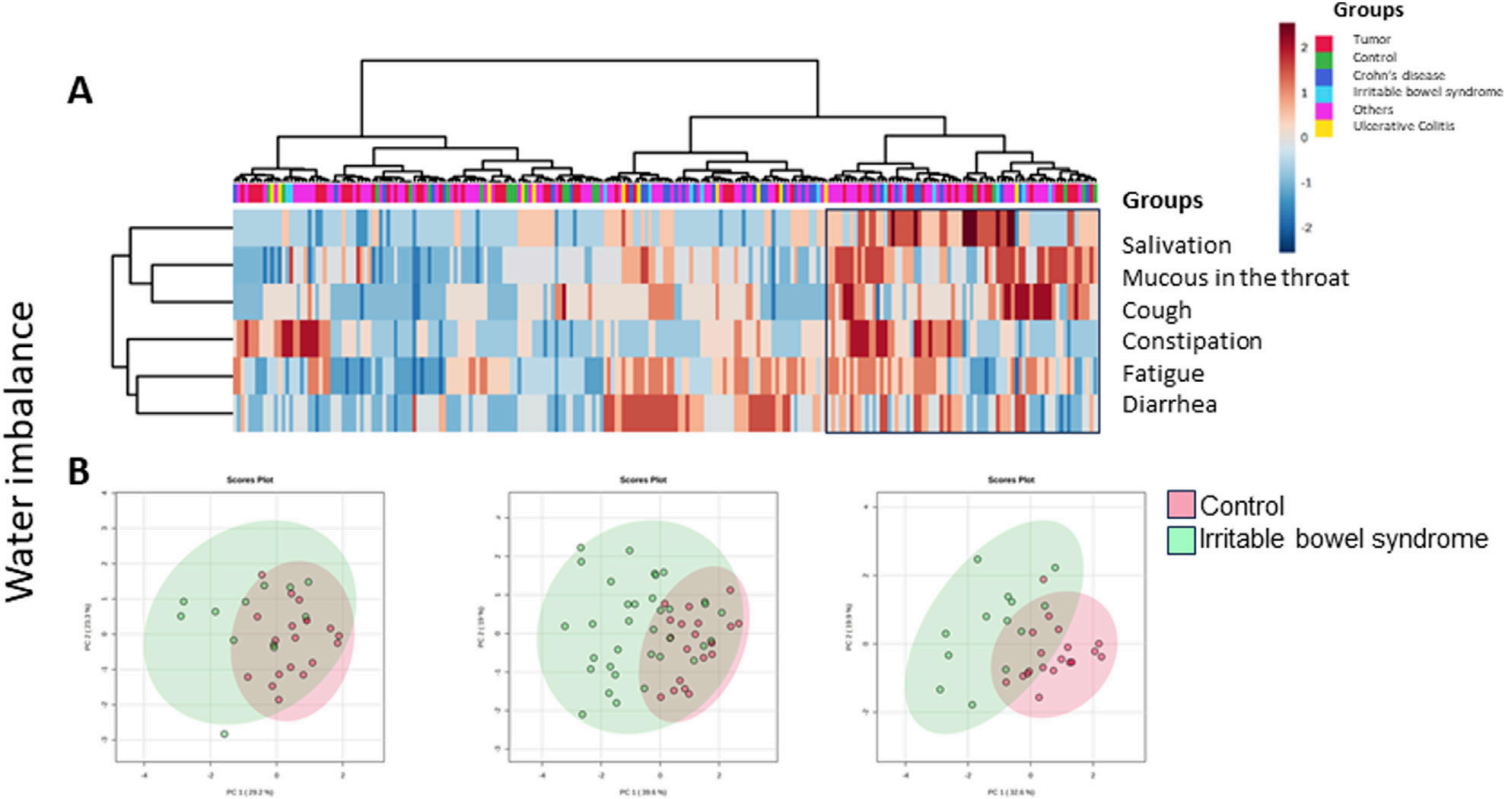
**(A)** Heatmap depicting Kampo-specific symptoms for fluid disturbance in the various groups of German patients and controls (MetaboAnalyst 5.0). Patients with typical symptoms are marked within the box. Although 72 patients showed symptoms of water imbalance, in none of the groups it became significant (two-sided Fisher’s exact test). **(B)** Primary component analysis of Kampo symptoms regarding water imbalance within different groups of patients (green) versus controls (red). From left: ulcerative colitis, Crohn’s disease, and irritable bowel syndrome (IBS). All three groups showed a wide scattering of symptoms when compared to the control group.

Figure 10 shows the heatmap for symptoms with fluid disturbance. The black box marks patients with elevated symptom load (Figure 10A). Within this symptomatic group (72 patients), 29.2% (n = 21) were tumor patients, 1.4% (n = 1) had ulcerative colitis, 12.5% (n = 9) had Crohn’s disease, 8.3% (n = 6) had IBS, 41.7% (n = 30) had other gastrointestinal complaints, and 6.9% (n = 5) were controls. IBS patients were the most affected; however, the data did not become significant when compared to the remaining participants (Table 3). Yet, primary component analysis showed a large scattering of patients with ulcerative colitis, Crohn’s disease, and IBS compared to the control group (Figure 10B).

## Discussion

Traditional medicinal systems, such as Japanese Kampo medicine, evolved in a time when the patients’ own reports of their condition and observable symptoms according to an evolving physical examination were the only diagnostic tools of medical doctors. The analysis of symptom scores gives a cluster of possible symptom combinations, which have been summarized within the following groups: mental health, general condition, sleep quality, pain sensation, skin/eye/nose/mouth problems, abdominal complaints, appetite, fecal habits, urination, and cold or warm sensation. Disease names such as *inflammatory bowel disease* (IBD) or *irritable bowel syndrome* (IBS) did not exist; instead, descriptions of the observed symptom complexes served as the basis for therapy. Based on these symptom combinations, the aim of this study was to evaluate whether the ancient clinical understanding of a patients’ current physiological situation—or diagnosis pattern (the Japanese term being “sho”)—and gastrointestinal diseases as they are classified today, correspond.

Of a total of 251 participants, 231 patients with gastrointestinal complaints and 20 controls were prospectively included. Almost a quarter had gastrointestinal tumors (23%) or inflammatory bowel disease (21%), more than one-third (41%) had other gastrointestinal complaints, and a minority (6.8%) reported IBS. All patients were healthy enough to visit our outpatient clinics. Male and female patients were well-balanced, with slightly more female participants (57.4%). The median age was 65 years. Patients with Crohn’s disease had the longest disease duration, with 45 years for one patient. Disease duration or chronic disease is known to be both a physical and a mental burden. Especially for IBD patients, a strong connection between mental and physical status has been reported (Kaplan et al., 2019; Araki et al., 2020), which is reflected in our study. Especially Crohn’s disease patients had high symptom scores in questions concerning mental health, i.e., the feeling of depression, anxiety, forgetfulness, fatigue, irritability, being easily frightened and mood swings, sleep quality, and pain. It has been proposed that signaling events from the gut modulate brain function, which is referred to as the gut–brain axis (Günther et al., 2021). In comparison, it was almost surprising to see that our tumor patients were doing rather well, even when compared to controls. The same was true for gastrointestinal complaints, appetite, fecal habits or cold sensation, where tumor patients showed the least symptoms, in spite of abdominal tumor resections.

As symptoms as well as disease acuity or chronicity may differ significantly from patient to patient, the description and evaluation of individual symptoms might be important for future individualization of treatment decisions and biomarker validation. The questionnaire could further aid to characterize the change in symptom patterns throughout therapy. The current study was the first step to validate the questionnaire in a German patient collective. We could demonstrate for the first time that a representative Kampo questionnaire can be successfully used in a German patient collective. We could further show that no separation was found when comparing German to Japanese patients.

Although significant differences within the patient groups were found, there was no clustering of individual symptoms and the various underlying diagnoses: Symptoms remained individual, regardless of the disease or origin of the patient. This is important to note as Western scores—such as the Clinical Disease Activity Index (CDAI) for IBD-patients or the UICC-tumor–node–metastasis (TNM) classification for tumor patients—do not comprise the individual body constitution. Daily living abilities—such as depicted by the Eastern Cooperative Oncology Group (ECOG) performance status or the Karnofsky index—are secondary to individual complaints as human beings are resilient and can cope over a long time. Our data show that symptom patterns underscore the individual patients’ complaints at a given time point and might offer a road to individualized therapy. In traditional medicines, this is the case: The treatment is tailored to the symptoms presented. The host’s condition and energy levels are highly valued (Yakubo et al., 2014). Instead of symptom patterns, modern diagnosis differentiates the various underlying diseases. Both systems supplement each other. Sometimes, treatment of the underlying disease resolves the reported symptoms, and in other cases, it does not. For instance, IBD patients can report joint pain even though mucosal healing has been obtained.

Our results anticipate that for the conception of future clinical studies, Western disease criteria alone might not be sufficient for patient recruitment. For Kampo remedies, this has already been shown. To give an example, a clinical study was performed for hot flushes in menopause, with the Japanese Kampo prescription Keishibukuryogan (桂枝茯苓丸). The inclusion criteria included vasomotor symptoms, as documented by a hot flush symptom diary (Plotnikoff et al., 2011). However, Keishibukuryogan is usually not only used for hot flushes alone but also for lower abdominal pain, menstruation disorders, cold sensitivity, shoulder stiffness, dizziness, skin eczema, and others (Eberhard, 2003; Terasawa, 1994). To filter those patients who would benefit from the prescription, these questions should have been considered. It can be seen similar to modern Western medicine trials, where the mutation of certain cancers (KRAS, BRAF, and c-KIT) or the combined positive score (CPS) for immune-checkpoint inhibitors need to be tested. In this context, it would be interesting to test in Western clinical studies whether slim, anxious patients with cold limbs and shallow sleep would profit similarly from the same remedy than patients with a better body constitution. We only know that—like for cancer patients—the better the body constitution, the better the outcome. It would further be exciting to see, whether “weaker” patients would benefit from a warming remedy (such as a ginger-containing formula or other, which improves appetite and blood circulation within the intestine) and would thus further profit from immune modulators/biologicals, and if so, which ones? Specific symptoms and clinical findings might further be linked such as the geographic tongue in celiac disease (i.e., distress of the immune system) and thus be related to specific biomarkers.

To make such studies possible, a combined effort has been made by a WHO initiative to extend the International Classification of Diseases (ICD) in version 11 to incorporate symptom-based features. It was adopted by the World Health Assembly in 05/2019 and took effect in 01/2022. After a flexible transition period of 5 years, ICD reporting will be exclusively by ICD-11 (WHO, 2019). It also includes traditional Kampo patterns such as energy (Qi)-deficiency, blood stasis and deficiency, and fluid disturbances (Wu et al., 2021; WHO, 2019). This is important since Kampo medicines can be used to support a weak body constitution as “*hozai*” (補剤), especially in the Western context of cachexia (Motoo and Cameron, 2022; Kuchta and Cameron, 2020), geriatrics (Takayama et al., 2018), and adjuvant cancer therapy (Cameron et al., 2018; Motoo and Cameron, 2022), settings in which Western pharmacotherapy alone has often proven powerless. There are attempts by the WHO to include a traditional questionnaire into their system (communication KW, WHO representative). Our questionnaire might be a primordial version. It also has a few shortcomings, which need to be resolved. In our analysis, symptom scores for the individual symptom blocks were obtained by adding the individual points per block, followed by division through the number of questions. Although this seems to be straight forward, it does not always differentiate between strong and weak patients, i.e., symptoms of increased appetite and loss of appetite, diarrhea and constipation. As a consequence, we classified our symptoms not only according to Western symptom blocks and heatmaps depicting the individual symptoms but also according to traditional symptom blocks, such as Qi-deficiency, blood stagnation, or fluid disturbance. As individual symptoms within traditional items might overlap, they are grouped together as symptom patterns. Furthermore, efforts are being made by working group 5 of ISO-TC-249 to standardize traditional Chinese vocabulary (https://www.iso.org/committee/598435.htm committee/598435.html).

Regarding traditional terms, approximately half of our patients had symptoms of Qi-deficiency. Even though otherwise our patients performed well, Qi-deficiency was found within 43% of the tumor group, 92% of patients with ulcerative colitis, 71% of patients with Crohn’s disease, and 62% of the IBS patients. Although Qi-deficiency is usually associated with the lack of vital energy, it is related to poor digestion and, as a consequence, nutrition. In traditional understanding, symptom patterns can point to a weakness according to the five-organ theory, where digestive functions would relate to the idea of “spleen”. This is supported by the results on spleen-related symptoms (data not shown), especially in Crohn’s disease patients (47%), followed by IBS patients (46%), with a large scattering of symptoms for all patients in comparison to controls. This implicates that the old theories still hold within modern analysis.

As for Qi-stagnation, similar results were obtained for patients with Crohn’s disease (59% showed Qi-stagnation) and patients with IBS (where 61% reported symptoms of Qi-stagnation), including inner unrest, sleeping problems, nervosity, palpitations, headache, nausea, and dizziness. As a consequence, we have to be aware that these symptoms are part of the underlying intestinal disease and not an annoying side-demeanour. According to traditional principles, they relate to liver imbalance, but in modern understanding, it might also be part of the aforementioned gut–brain axis (Günther et al., 2021).

Deficiency of blood circulation was observed in more than 50% of all patients and in more than 60% of patients with Crohn’s disease and IBS. Even controls showed corresponding symptoms in 45%. Symptoms include—among others—tired eyes, rings underneath the eyes, headache, menstruation disorders or early menopause, constipation, and cold sensation.

In view of these results, prevention can be done before overt disease occurs. Awareness of symptoms and signs can pinpoint to a subgroup of people without apparent disease who are not completely healthy. This is in line with the Kampo concept of *Mibyo* (未病), the pre-pathological condition in which the physiological state has already deviated from the optimal healthy condition and is in the process of deteriorating into a pathological state (Uto et al., 2018). This concept was first described in the Huangdi Neijing (黄帝内経 ‐ *Koteinaikei*) and the Qianjin Yaofang ([備急]千金要方 ‐ *Senkinyoho*), two prominent textbooks of ancient Chinese medicine. For didactical purposes and to enhance patient’ compliance, mibyo has been recently refered to as “ME-BYO”. A concept incorporating the English word “me” to emphasize that “me,” the patient him-/herself can actively contribute to prevent overt disease, and doctors should be aware of it (Uto et al., 2018). Based on this reasoning, the traditional concept has recently been modified into the “ME-BYO index” (Nakamura et al., 2023).

Blood stagnation (*Oketsu*/瘀血) or fluid disturbance (*Suidoku*/水毒) played a minor role in our patient collective. However, in patients with inflammatory bowel disease or IBS, symptoms showed a large scattering when compared to controls.

Our results confirm that Kampo medicine does not need a specific disease setting. It remains symptom-oriented, a benefit, rather than an obstacle. It also offers answers to symptoms or symptom complexes which remain within a scientific void, such as the leaky-gut syndrome with intestinal lymphangiectasia and concurrent edema, long-COVID with mnestic impairment or chronic fatigue syndrome, and the role of the microbiome. A routine in describing symptoms of patients might help to gather insight into complex diseases and, finally, their mechanism of action. Although symptoms might be perceived differently, patterns of symptoms evolved and, in traditional understanding, need adjustment of medication within an individual patient.

In our analysis, we could extract a group of patients showing symptoms typical for the *hozai* (補剤) prescription *Juzentaihoto*, with significantly matching results for Crohn’s disease patients. As in all other entities, our outpatient tumor patients performed significantly better than the remaining patient collective, pointing to a good general condition, probably because of a rather short disease duration and primarily healthy individuals. Similarly, *Hochuekkito* suited 32% of Crohn’s disease patients. In clinical routine, however, it is also employed to strengthen tumor patients (Cameron et al., 2018; Motoo and Cameron, 2022). Again, our tumor patients had a better body constitution (*sho*) and were not as weak as patients, in a palliative setting who might benefit from *Hochuekkito*. Hence, the disease stage and symptom pattern need to be accounted for. *Rikkunshito*, expectedly, suited 22% of patients with general gastrointestinal problems including patients with dyspepsia and upper gastrointestinal complaints (Reißenweber-Hewel et al., 2023). It is known that *Rikkunshito* improves gastric motility dysfunction with impaired adaptive relaxation and delayed gastric emptying, gastric hypersensitivity, and anorexia via facilitating ghrelin secretion (Oka et al., 2014). It also exhibits anti-stress effects, i.e., it attenuates stress-induced exacerbation of gastric sensation and anorexia and sympathetic activation (Motoo and Cameron, 2022; Oka et al., 2014). In our study, 47% of Crohn’s disease patients had typical symptoms fitting the prescription. Nevertheless, 68% of patients with Crohn’s disease and 77% of patients with IBS as well as 47% of the patients with other GI complaints also suited *Keishikashakuyakuto*, a prescription which is used in rather weak patients with abdominal distention and cramps or pain (Oka et al., 2014). This reflects how important it is to check the related symptom patterns (kuketsu) and tailor traditional therapy accordingly. It might further help to understand the underlying physiological processes as the chemical structures of the individual plant components are known, and their activity can be tested *in silico*, *in vitro*, and *in vivo*. As time is critical in modern medicine, questionnaires and their analysis—if possible, by means of artificial intelligence—might help.

Kampo physical examination complements disease pattern. It includes abdominal palpation, tongue, and pulse diagnosis. These exams are fitting to our Western physical examination. They have just been forgotten in the daily routine practice. In pediatrics, for instance, tongue diagnosis is still performed and in adults we describe a raspberry tongue (in liver cirrhosis patients) without coating, and a geographic tongue in patients with a weak immune system (Siegenthaler, 2005).

Physical examination according to Kampo criteria was done in one-third of our patients (n = 80). A bloated abdomen was described in half of the IBS patients, while tension of the rectus abdominal muscles was documented mainly in Crohn’s disease patients; however, numbers were small. Tension of the rectus abdominal muscles would be a feature for the Kampo prescription *Keishikashakuyakuto*.

As for tongue diagnosis, coating was only found in a minor number (17.5%) of all patients, suggesting that they were rather weak whilst coping in daily life. Tooth imprints were documented in more than a quarter of the patients, featuring patients with ulcerative colitis and IBS; however, again their numbers were negligible. As a sign of exertion, Crohn’s disease and IBS patients showed red dots at the tips of their tongue. Venous congestion as a sign of blood stagnation was found in tumor and inflammatory bowel disease patients; however, it was not found in IBS patients. Further studies should be done in this regard.

A rather weak body constitution is also mirrored by the deep pulse, as reported in 50% of the patients. A tense pulse was found mostly in patients with Crohn’s disease or IBS. Only in 20% a strong pulse was reported, mostly in tumor patients, which complies with their tentatively better results within the symptom scores.

It is obvious that this study can only be a start for generating awareness for preclinical and accompanying symptoms and signs also in a Western patient collective. A detailed anamnesis and physical examination help understand the individual patients’ pattern diagnosis and should not be forgotten in modern times.

## Conclusion

Our data summarize—for the first time—information on a traditional symptom questionnaire in a Western medical system with German patients. Even though the control and Japanese patient collectives were small for comparison, we found that the questionnaire is applicable in German patients and warrants further study. The separation of symptoms according to diseases remains difficult, pointing out that patients should not solely be visualized within the frame of their disease but according to the various symptoms they present. The individual body constitution and symptom patterns need to be considered even in a Western medical context, especially in view of its apparent individualization. Not only in view of dosage the patient’s constitution should be taken into account especially for chemotherapy. Although the performance status of the Eastern Cooperative Oncology Group (ECOG) or the Karnofsky index is being noted, the body mass index (BMI) is still mostly considered for the calculation of chemotherapy dosing. For clinical studies, inclusion criteria according to symptom patterns could be of help. For clinical trials with herbal remedies, traditional anamnesis and examination are necessary.

As in ancient times, the physician should be aware of the individual patient’s complaints and treat him accordingly. This might not only guide the treatment but also aid in patient–doctor interaction. As in Japan, this questionnaire—in a simplified version—might be of help, especially for patients with complex symptom patterns.

To summarize, we showed that a) the questionnaire is valid for the analysis of a patients’ body constitution at a given time point—also in German patients; b) this is independent of the underlying disease. We suggest that the resulting individual pattern diagnosis should be taken into account to help treatment individualization or the finding of biomarkers in the future. We propose that our findings anticipate clinical studies where a shortened version of the questionnaire can be applied before and during treatment.

## Data Availability

The original contributions presented in the study are included in the article/Supplementary Material; further inquiries can be directed to the corresponding author.

## Supplementary material

SUPPLEMENTARY FIGURE 1 Heatmap of the various groups: tumor, UC, CD, IBS, and others in view of symptom expression. The upper line shows the distribution of various groups, now regrouped from Figure 5 according to disease. No clustering of patients/controls and symptom score was observed (MetaboAnalyst 5.0).
