# Supplementary material for: Traditional clinical symptoms and signs: Kampo pattern diagnosis in modern gastrointestinal disease
Source: Front Pharmacol. 2024 Sep 27;15:1426491. doi: 10.3389/fphar.2024.1426491 (PMC11472708; doi:10.3389/fphar.2024.1426491)
Supplement: Supplementary file 1 [file DataSheet2.PDF]

Abt. Gastroenterologie und
Allg. Innere Medizin
Chefärztin: PD Dr. med. S. Cameron
T 05541/996-718 F 05541/996-447

Klinikum Hann. Münden
Vogelsang 105, 34346 Hann. Münden

Klinik für Gastroenterologie und GI-Onkologie
Chefarzt: Prof. Dr. med. V. Ellenrieder
T 0551/39-66301

Universitätsmedizin Göttingen
Robert-Koch-Str. 40, 37075 Göttingen

アンケート調査

漢方医学的な体質の評価

性別： ☐男性 ☐女性

年齢: 歳

胃腸疾患の病歴: ☐ クローン病 ☐ 潰瘍性大腸炎 ☐ 腫瘍
☐ 過敏性腸症候群 ☐ その他（______________）

病気の期間： 年 月

それ以外の病歴: ☐ 心臓 ☐ 肺 ☐ 腎臓
☐ その他（______________________________）

現在、漢方薬を服用していますか？: はい ☐ いいえ

「はい」と答えた場合に、どのような漢方薬を服用していますか？

....

....

現代の状態に最も近い答にチェックを入れてください。

精神状態:

うつっぽい	☐ まったくない	☐めったにない	☐たまに	☐頻繁に
不安な気持ちになる	☐ まったくない	☐めったにない	☐たまに	☐頻繁に
忘れっぽい	☐ まったくない	☐めったにない	☐たまに	☐頻繁に
疲れやすい	☐ まったくない	☐めったにない	☐たまに	☐頻繁に
イライラする	☐ まったくない	☐めったにない	☐たまに	☐頻繁に
すぐに恐怖を感じる	☐ まったくない	☐めったにない	☐たまに	☐頻繁に
気分の上下が激しい	☐ まったくない	☐めったにない	☐たまに	☐頻繁に

全身の状態:

よく汗をかく	☐全身	☐顔	☐手足	
	☐寝汗			
汗の減少	☐まったくない	☐めったにない	☐たまに	☐頻繁に
水がたまる	☐体	☐脚		
手足の不快な感覚（ムズムズする、まひ等）				
	☐まったくない	☐めったにない	☐たまに	☐頻繁に
立ち上がったときのめまい/目の前が黒くなる				
	☐まったくない	☐めったにない	☐たまに	☐頻繁

睡眠：

精神的に落ち着かない	☐まったくない	☐めったにない	☐たまに	☐頻繁に
眠りにつきにくい	☐まったくない	☐めったにない	☐たまに	☐頻繁に
夜中に目が覚める	☐まったくない	☐めったにない	☐たまに	☐頻繁に
よく夢を見る	☐まったくない	☐めったにない	☐たまに	☐頻繁に
日中眠たくなる	☐まったくない	☐めったにない	☐たまに	☐頻繁に

**痛みを感じる：**				
小さな関節（指等）	□まったくない	□めったにない	□たまに	□頻繁に
大きな関節(膝、股関節等)	□まったくない	□めったにない	□たまに	□頻繁に
背中	□まったくない	□めったにない	□たまに	□頻繁に
筋肉痛	□まったくない	□めったにない	□たまに	□頻繁に

**皮膚：**				
乾燥している	□まったくない	□めったにない	□たまに	□頻繁に
潤いのある	□まったくない	□めったにない	□たまに	□頻繁に
痒みがある	□まったくない	□めったにない	□たまに	□頻繁に
爪がわれている	□まったくない	□めったにない	□たまに	□頻繁に
抜け毛がある（髪の毛をとかす時など）		□頭　□皮膚		

**頭：**				
頭痛がする	□まったくない	□めったにない	□たまに	□頻繁に
ぼうっとしている	□まったくない	□めったにない	□たまに	□頻繁に
乗り物酔いがある（例：船旅での目眩 / 吐き気）				
	□まったくない	□めったにない	□たまに	□頻繁に
その他	□暑い	□寒気い		

**目：**				
目が疲れている	□まったくない	□めったにない	□たまに	□頻繁に
目が赤くなる	□まったくない	□めったにない	□たまに	□頻繁に
目の下に隈がある	□まったくない	□めったにない	□たまに	□頻繁に

鼻：

くしゃみがよく出る	□まったくない	□めったにない	□たまに	□頻繁に
鼻水が出る	□水っぽい	□粘り気がある		
痰がでる	□まったくない	□めったにない	□たまに	□頻繁に
鼻で息をするのが困難である / 鼻がつまる				
	□まったくない	□めったにない	□たまに	□頻繁に
その他	□鼻が乾く　□鼻血			

口：

口がかわく	□まったくない	□めったにない	□たまに	□頻繁に
苦い味がする	□まったくない	□めったにない	□たまに	□頻繁に
唾液の分泌が良くない	□まったくない	□めったにない	□たまに	□頻繁に
味覚が変化する	□まったくない	□めったにない	□たまに	□頻繁に
舌の痛みを感じる	□まったくない	□めったにない	□たまに	□頻繁に
よく口内炎を起こす	□まったくない	□めったにない	□たまに	□頻繁に
唇が乾いている	□まったくない	□めったにない	□たまに	□頻繁に

耳：

耳鳴りがする	□まったくない	□めったにない	□たまに	□頻繁に
耳が聞こえにくい	□まったくない	□めったにない	□たまに	□頻繁に

首：

喉の痛み	□まったくない	□めったにない	□たまに	□頻繁に
喉が痞えた感じがする	□まったくない	□めったにない	□たまに	□頻繁に
喉の乾燥	□まったくない	□めったにない	□たまに	□頻繁に
声がかすれる	□まったくない	□めったにない	□たまに	□頻繁に

胸部：

ねばねばした淡が出る				
	☐まったくない	☐めったにない	☐たまに	☐頻繁に
咳が出る	☐まったくない	☐めったにない	☐たまに	☐頻繁に
ぜいぜいする	☐まったくない	☐めったにない	☐たまに	☐頻繁に
息切れがする	☐まったくない	☐めったにない	☐たまに	☐頻繁に
動悸がする	☐まったくない	☐めったにない	☐たまに	☐頻繁に
胸の痛みがある	☐まったくない	☐めったにない	☐たまに	☐頻繁に
その他	☐腹痛	☐胸焼け		

腹部：

げっぷがでる	☐まったくない	☐めったにない	☐たまに	☐頻繁に
吐き気がする	☐まったくない	☐めったにない	☐たまに	☐頻繁に
嘔吐する	☐まったくない	☐めったにない	☐たまに	☐頻繁に
腹痛がある	☐上腹部	☐下腹部	☐腹部全体	☐移動する
お腹が膨らむ	☐まったくない	☐めったにない	☐たまに	☐頻繁に
お腹が鳴る	☐まったくない	☐めったにない	☐たまに	☐頻繁に
消化の問題がある	☐まったくない	☐めったにない	☐たまに	☐頻繁に

食欲：

食欲の減退	☐まったくない	☐めったにない	☐たまに	☐頻繁に
食欲の増加	☐まったくない	☐めったにない	☐たまに	☐頻繁に
食事を楽しめない	☐まったくない	☐めったにない	☐たまに	☐頻繁に

どのような食べ物/飲み物が好きですか（複数の選択肢があります）：

☐甘いもの	☐塩辛いもの	☐酸っぱいもの	☐辛いもの
☐脂っこいもの	☐冷たいもの	☐温かいもの	
☐肉/魚	☐野菜/果物	☐乳製品	☐炭酸飲料

嫌いな食べ物や飲み物（下線の上に書いてください）：

シナモン、コショウ、セロリ、生姜、さつまいも、ごま　等：________________________________

食生活・食習慣：

主食（朝食、昼食、夕食）	☐きちんと食べる	☐食事を抜かすことがある
おやつを食べる	☐はい	☐いいえ
就寝前のおやつを食べる	☐はい	☐いいえ

排便習慣：

よく便秘になる	☐まったくない	☐めったにない	☐たまに	☐頻繁に
よく下痢をする	☐まったくない	☐めったにない	☐たまに	☐頻繁に
便秘と下痢を繰り返す	☐まったくない	☐めったにない	☐たまに	☐頻繁に
痔がある	☐まったくない	☐めったにない	☐たまに	☐頻繁に

尿：

24 時間以内に何回位排尿しますか？ ☐1-3 回　☐4-6 回　☐7-9 回　☐10 回以上

夜に何回トイレに行きますか？　＿＿＿＿ 回

尿の量	☐多い	☐普通	☐少ない
排尿に問題がありますか？	☐はい	☐いいえ	
排尿時に痛みがありますか？	☐はい	☐いいえ	
尿漏れをしますか？	☐はい	☐いいえ	

体の硬さ / 動かしにくさ：

首がこる	☐まったくない	☐めったにない	☐たまに	☐頻繁に
肩こりがある	☐まったくない	☐めったにない	☐たまに	☐頻繁に
腰痛がある	☐まったくない	☐めったにない	☐たまに	☐頻繁に

寒気 / 悪寒を感じる：

手	☐まったくない	☐めったにない	☐たまに	☐頻繁に
足	☐まったくない	☐めったにない	☐たまに	☐頻繁に
お尻	☐まったくない	☐めったにない	☐たまに	☐頻繁に
お腹	☐まったくない	☐めったにない	☐たまに	☐頻繁に
背中	☐まったくない	☐めったにない	☐たまに	☐頻繁に
全身に	☐まったくない	☐めったにない	☐たまに	☐頻繁に

月経について：

最初の月経は何歳の時でしたか？ ________ 歳

妊娠の可能性はありますか？ ☐はい ☐いいえ

生理中に鎮痛剤を服用していますか？ ☐はい ☐いいえ

妊娠した子どもの数： ________________ 人

出産した子どもの数： ________________ 人

閉経：____________ 歳

不正出血がありますか？ ☐まったくない ☐めったにない ☐たまに ☐頻繁に

生理は重いほうですか？ ☐重い ☐中程度 ☐弱い

月経前にイライラしますか？（PMS）

☐まったくない ☐めったにない ☐たまに ☐頻繁に

ご協力どうもありがとうございました！
