# Supplementary figures and images for: Traditional clinical symptoms and signs: Kampo pattern diagnosis in modern gastrointestinal disease
Source: Front Pharmacol. 2024 Sep 27;15:1426491. doi: 10.3389/fphar.2024.1426491 (PMC11472708; doi:10.3389/fphar.2024.1426491)

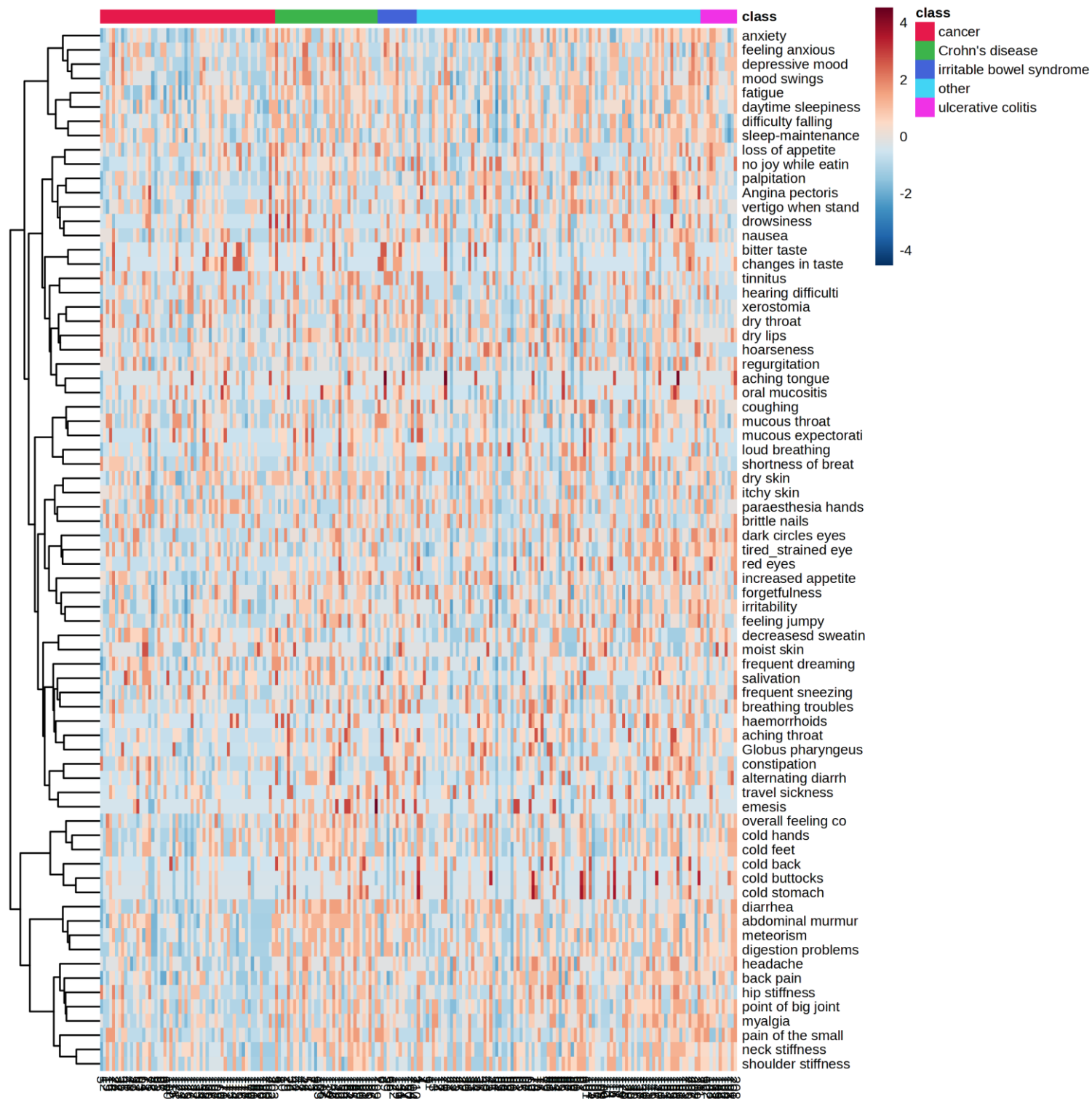

Supplement: Supplementary file 2 [file DataSheet4.PDF]

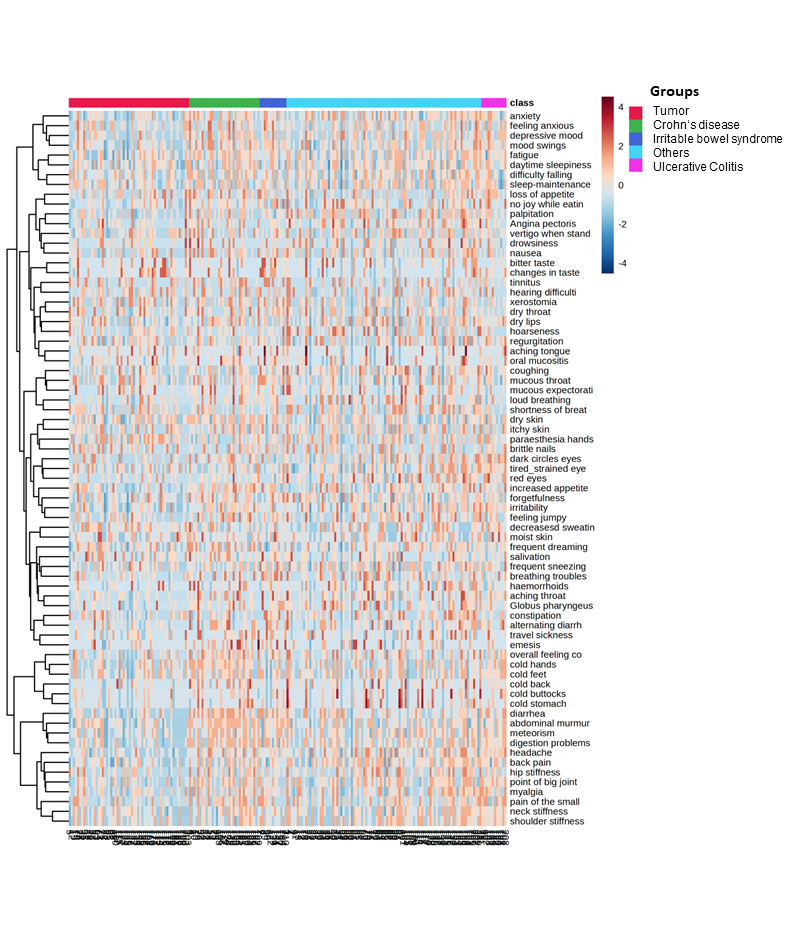

Supplement: Supplementary file 3 [file Image1.tif]
