# Supplementary material for: Traditional clinical symptoms and signs: Kampo pattern diagnosis in modern gastrointestinal disease
Source: Front Pharmacol. 2024 Sep 27;15:1426491. doi: 10.3389/fphar.2024.1426491 (PMC11472708; doi:10.3389/fphar.2024.1426491)
Supplement: Supplementary file 4 [file DataSheet3.PDF]

Abt. Gastroenterologie und
Allg. Innere Medizin
Chefärztin: PD Dr. med. S. Cameron
T 05541/996-718 F 05541/996-447

Klinikum Hann. Münden
Vogelsang 105, 34346 Hann. Münden

Klinik für Gastroenterologie und GI-Onkologie
Chefarzt: Prof. Dr. med. V. Ellenrieder
T 0551/39-66301

Universitätsmedizin Göttingen
Robert-Koch-Str. 40, 37075 Göttingen

Questionnaire

To evaluate the body constitution in the context of Japanese Kampo medicine

Sex: □ male □ female

Age: …..……… years

Gastrointestinal disease: □ Crohn's disease □ Ulcerative Colitis □ Cancer

□ Irritable Colon □ Other

Duration of disease: ………………………………… **years,** for instance 1 ½ years

Other diseases: □ heart □ lung □ kidney

Do you currently use herbal preparations: □ Yes □ No

If yes: which ones?

………..

……

Please mark what fits best to your current condition.

State of mind:

Depressive mood	□ not at all	□ rarely	□ occasionally	□ common
Anxiety	□ not at all	□ rarely	□ occasionally	□ common
Forgetfulness	□ not at all	□ rarely	□ occasionally	□ common
Fatigue	□ not at all	□ rarely	□ occasionally	□ common
Irritability	□ not at all	□ rarely	□ occasionally	□ common
Easily Startled	□ not at all	□ rarely	□ occasionally	□ common
Mood Swings	□ not at all	□ rarely	□ occasionally	□ common

General condition:

Frequent sweating	□ all over the body		□ face	□ hands and feet
	□ night sweat			
Decreased sweating	□ not at all	□ rarely	□ occasionally	□ common
Fluid retention	□ body	□ legs		

Paresthesia in hands and feet (such as tingling or numbness)

□ not at all □ rarely □ occasionally □ common

Vertigo when standing up / everything becomes dark when suddenly standing up?

□ not at all □ rarely □ occasionally □ common

Sleep:

Inner unrest	□ not at all	□ rarely	□ occasionally	□ common
Problems falling asleep	□ not at all	□ rarely	□ occasionally	□ common
Problems sleeping	□ not at all	□ rarely	□ occasionally	□ common
Frequent dreams	□ not at all	□ rarely	□ occasionally	□ common
Daytime sleepiness	□ not at all	□ rarely	□ occasionally	□ common

Pain

Small joints (such as fingers)	□ not at all	□ rarely	□ occasionally	□ common
Large joints (such as knee, hips)	□ not at all	□ rarely	□ occasionally	□ common
Back	□ not at all	□ rarely	□ occasionally	□ common
Muscle pain	□ not at all	□ rarely	□ occasionally	□ common

Skin

Dry	□ not at all	□ rarely	□ occasionally	□ common
Moist	□ not at all	□ rarely	□ occasionally	□ common
Itchy	□ not at all	□ rarely	□ occasionally	□ common
Brittle nails	□ not at all	□ rarely	□ occasionally	□ common
Hair loss (for instance during combing)		□ head	□ skin	

Head

Headache	□ not at all	□ rarely	□ occasionally	□ common
Drowsiness	□ not at all	□ rarely	□ occasionally	□ common
Motion sickness (such as vertigo/nausea during shipping)				
	□ not at all	□ rarely	□ occasionally	□ common
Other	□ feeling of heat		□ sensation of cold	

Eyes

Tired/strained eyes	□ not at all	□ rarely	□ occasionally	□ common
Red eyes	□ not at all	□ rarely	□ occasionally	□ common
Rings underneath the eyes	□ not at all	□ rarely	□ occasionally	□ common

Nose

Frequent sneezing	□ not at all	□ rarely	□ occasionally	□ common
Rhinorrhoea	□ watery	□ slabby/mucoid		
Mucus in the throat	□ not at all	□ rarely	□ occasionally	□ common
Problems to breathe through the nose				
	□ not at all	□ rarely	□ occasionally	□ common
Other	□ dry nose	□ nosebleed		

Mouth

Dry mouth	□ not at all	□ rarely	□ occasionally	□ common
Bitter taste	□ not at all	□ rarely	□ occasionally	□ common
Salivation	□ not at all	□ rarely	□ occasionally	□ common
Changed taste	□ not at all	□ rarely	□ occasionally	□ common
Painful tongue	□ not at all	□ rarely	□ occasionally	□ common
Frequent mouth inflammation	□ not at all	□ rarely	□ occasionally	□ common
Dry lips	□ not at all	□ rarely	□ occasionally	□ common

Ears

Tinnitus	□ not at all	□ rarely	□ occasionally	□ common
Auditory problems	□ not at all	□ rarely	□ occasionally	□ common

Neck

Sore throat	□ not at all	□ rarely	□ occasionally	□ common
Globus sensation	□ not at all	□ rarely	□ occasionally	□ common
Dry throat	□ not at all	□ rarely	□ occasionally	□ common
Hoarse voice	□ not at all	□ rarely	□ occasionally	□ common

Chest area

Mucous phlegm	□ not at all	□ rarely	□ occasionally	□ common
Cough	□ not at all	□ rarely	□ occasionally	□ common
Audible breathing	□ not at all	□ rarely	□ occasionally	□ common
Short of breath	□ not at all	□ rarely	□ occasionally	□ common
Heart palpitations	□ not at all	□ rarely	□ occasionally	□ common
Chest pain	□ not at all	□ rarely	□ occasionally	□ common
Other	□ gastric ache	□ heartburn		

Abdomen

Belching	□ not at all	□ rarely	□ occasionally	□ common
Nausea	□ not at all	□ rarely	□ occasionally	□ common
Vomiting	□ not at all	□ rarely	□ occasionally	□ common
Abdominal pain	□ upper abdomen	□ lower abdomen	□ general	
Distended abdomen	□ not at all	□ rarely	□ occasionally	□ common
Abdominal murmur	□ not at all	□ rarely	□ occasionally	□ common
Digestive problems	□ not at all	□ rarely	□ occasionally	□ common

Appetite:

Loss of appetite	□ not at all	□ rarely	□ occasionally	□ common
Increased appetite	□ not at all	□ rarely	□ occasionally	□ common
can’t enjoy eating	□ not at all	□ rarely	□ occasionally	□ common

What kind of food/drinks do you like? (multiple choices are possible):

□ sweet □ salty □ sour □ spicy

□ fatty □ cold □warm

□ meat/fish □ vegetables/fruit □ dairy products □ lemonade

Food and drinks you do not like (Please underline):
cinnamon, pepper, celery, ginger, japanese yam root, sesame: ………………………………

Dietary habits:

Large meals (breakfast, lunch, dinner)	□ regularly	□ irregularly
Snacks	□ yes	□ no
Snack before going to bed	□ yes	□ no

Fecal habits

Often constipation/congestion	□ not at all	□ rarely	□ occasionally	□ common
Often diarrhoea	□ not at all	□ rarely	□ occasionally	□ common
Alternation of constipation and diarrhoea				
	□ not at all	□ rarely	□ occasionally	□ common
Haemorrhoids	□ not at all	□ rarely	□ occasionally	□ common

Urine:

How often to you need to urinate within 24 h? □ 1-3 □ 4-6 □ 7-9 □ >10

How often do you need to urinate during the night? ……………………

Quantity of urine:	□ much	□ normal	□ few
Do you have problems with urination?	□ yes	□ no	
Do you have pain during urination?	□ yes	□ no	
Do you have urinary incontinence?	□ yes	□ no	

Stiffness

Neck	□ not at all	□ rarely	□ occasionally	□ common
Shoulders	□ not at all	□ rarely	□ occasionally	□ common
Hips	□ not at all	□ rarely	□ occasionally	□ common

Sensation of cold/shivers

Hands	□ not at all	□ rarely	□ occasionally	□ common
Feet	□ not at all	□ rarely	□ occasionally	□ common
Bottom	□ not at all	□ rarely	□ occasionally	□ common
Belly	□ not at all	□ rarely	□ occasionally	□ common
Back	□ not at all	□ rarely	□ occasionally	□ common
General	□ not at all	□ rarely	□ occasionally	□ common

Overview of menstruation

Your first menstruation was in the age of: …………….. years

Is there a possibility for pregnancy? □ yes □ no

Do/Did you use pain killers during your period? □ yes □ no

Number of children: ……………………

Menopause in the age of: ……………….. years

Irregular menstruation □ not at all □ rarely □ occasionally □ common

Quantity of menstruation □ strong □ medium strength □ weak

Irritability before menstruation (PMS)

□ not at all □ rarely □ occasionally □ common

How do you feel about the questionnaire:

□ useful □ less useful □ not useful

Thank you for your participation!
