# Supplementary material for: Traditional clinical symptoms and signs: Kampo pattern diagnosis in modern gastrointestinal disease
Source: Front Pharmacol. 2024 Sep 27;15:1426491. doi: 10.3389/fphar.2024.1426491 (PMC11472708; doi:10.3389/fphar.2024.1426491)
Supplement: Supplementary file 5 [file DataSheet1.PDF]

Abt. Gastroenterologie und
Allg. Innere Medizin
Chefärztin: PD Dr. med. S. Cameron
T 05541/996-718 F 05541/996-447

Klinikum Hann. Münden
Vogelsang 105, 34346 Hann. Münden

Klinik für Gastroenterologie und GI-Onkologie
Chefarzt: Prof. Dr. med. V. Ellenrieder
T 0551/39-66301

Universitätsmedizin Göttingen
Robert-Koch-Str. 40, 37075 Göttingen

Fragebogen

Zur Evaluation der Körperkonstitutionen im Rahmen der japanischen Kampo-Medizin

Geschlecht: □ männlich □ weiblich

Alter: …………. Jahre

Gastrointestinale Erkrankung: □ M. Crohn □ Colitis Ulcerosa □ Tumor
□ Reizdarm □ Anderes

Erkrankungsdauer: ………………………………… **Jahre**, z.B. 1 ½ Jahre

Weitere Erkrankungen an: □ Herz □ Lunge □ Niere

Nehmen Sie aktuell pflanzliche Präparate: □ Ja □ Nein

Wenn ja, welche?

……….

………..

Bitte kreuzen Sie an, was am ehesten Ihrem Zustand entspricht.

Geistige Verfassung:

Depressive Stimmungslage	□ gar nicht	□ selten	□ gelegentlich	□ häufig
Ängstliche Stimmungslage	□ gar nicht	□ selten	□ gelegentlich	□ häufig
Vergesslichkeit	□ gar nicht	□ selten	□ gelegentlich	□ häufig
Fatigue/Erschöpfung	□ gar nicht	□ selten	□ gelegentlich	□ häufig
Reizbarkeit	□ gar nicht	□ selten	□ gelegentlich	□ häufig
anfällig für Schreckreaktionen	□ gar nicht	□ selten	□ gelegentlich	□ häufig
Emotionale Schwankungen	□ gar nicht	□ selten	□ gelegentlich	□ häufig

Allgemeinbefinden:

Häufiges Schwitzen □ am ganzen Körper □ Gesicht □ Hände und Füße

□ Nachtschweiß

Vermindertes Schwitzen	□ gar nicht	□ selten	□ gelegentlich	□ häufig
Wassereinlagerungen	□ Körper	□ Beine		

Missempfindungen in Händen und Füßen (z.B. Kribbeln, Taubheitsgefühl)

□ gar nicht □ selten □ gelegentlich □ häufig

Schwindel bei dem Aufstehen/Schwarzwerden vor Augen

□ gar nicht □ selten □ gelegentlich □ häufig

Schlaf:

Innere Unruhe	□ gar nicht	□ selten	□ gelegentlich	□ häufig
Probleme beim Einschlafen	□ gar nicht	□ selten	□ gelegentlich	□ häufig
Probleme beim Durchschlafen	□ gar nicht	□ selten	□ gelegentlich	□ häufig
häufiges Träumen	□ gar nicht	□ selten	□ gelegentlich	□ häufig
Tagesmüdigkeit	□ gar nicht	□ selten	□ gelegentlich	□ häufig

Schmerzen

Kleine Gelenke (z.B. Finger)	□ gar nicht	□ selten	□ gelegentlich	□ häufig
Große Gelenke (z.B. Knie, Hüfte)	□ gar nicht	□ selten	□ gelegentlich	□ häufig
Rücken	□ gar nicht	□ selten	□ gelegentlich	□ häufig
Muskelschmerzen	□ gar nicht	□ selten	□ gelegentlich	□ häufig

Haut

Trocken	□ gar nicht	□ selten	□ gelegentlich	□ häufig
Feucht	□ gar nicht	□ selten	□ gelegentlich	□ häufig
Juckend	□ gar nicht	□ selten	□ gelegentlich	□ häufig
Brüchige Nägel	□ gar nicht	□ selten	□ gelegentlich	□ häufig
Haarverlust (z.B. beim Kämmen)		□ Kopf	□ Haut	

Kopf

Kopfschmerzen	□ gar nicht	□ selten	□ gelegentlich	□ häufig
Benommenheit	□ gar nicht	□ selten	□ gelegentlich	□ häufig
Reisekrankheit (z.B. Schwindel/Übelkeit während einer Schifffahrt)				
	□ gar nicht	□ selten	□ gelegentlich	□ häufig
Anderes	□ Hitzegefühl		□ Kältegefühl	

Augen

Müde/angestrengte Augen	□ gar nicht	□ selten	□ gelegentlich	□ häufig
Rote Augen	□ gar nicht	□ selten	□ gelegentlich	□ häufig
Ringe unter den Augen	□ gar nicht	□ selten	□ gelegentlich	□ häufig

Nase

Häufiges Niesen	□ gar nicht	□ selten	□ gelegentlich	□ häufig
Nasenausfluss	□ wässrig	□ Zäh/schleimig		
Schleim im Rachen	□ gar nicht	□ selten	□ gelegentlich	□ häufig
Schwierigkeiten beim Atmen durch die Nase/ Schwellung				
	□ gar nicht	□ selten	□ gelegentlich	□ häufig
Weiteres	□ trockene Nase	□ Nasenbluten		

Mund

Trockener Mund	□ gar nicht	□ selten	□ gelegentlich	□ häufig
Bitterer Geschmack	□ gar nicht	□ selten	□ gelegentlich	□ häufig
Speichelfluss	□ gar nicht	□ selten	□ gelegentlich	□ häufig
Veränderter Geschmack	□ gar nicht	□ selten	□ gelegentlich	□ häufig
Schmerzhafte Zunge	□ gar nicht	□ selten	□ gelegentlich	□ häufig
Häufige Mundentzündungen	□ gar nicht	□ selten	□ gelegentlich	□ häufig
Trockene Lippen	□ gar nicht	□ selten	□ gelegentlich	□ häufig

Ohren

Tinnitus	□ gar nicht	□ selten	□ gelegentlich	□ häufig
Hörschwierigkeiten	□ gar nicht	□ selten	□ gelegentlich	□ häufig

Hals

Halsschmerzen	□ gar nicht	□ selten	□ gelegentlich	□ häufig
Globusgefühl/Kloßgefühl	□ gar nicht	□ selten	□ gelegentlich	□ häufig
trockene Kehle	□ gar nicht	□ selten	□ gelegentlich	□ häufig
Heisere/raue Stimme	□ gar nicht	□ selten	□ gelegentlich	□ häufig

Brustraum

Schleimiger Auswurf	□ gar nicht	□ selten	□ gelegentlich	□ häufig
Husten	□ gar nicht	□ selten	□ gelegentlich	□ häufig
Hörbares/lautes Atmen	□ gar nicht	□ selten	□ gelegentlich	□ häufig
Kurzatmigkeit	□ gar nicht	□ selten	□ gelegentlich	□ häufig
Herzklopfen	□ gar nicht	□ selten	□ gelegentlich	□ häufig
Brustschmerz	□ gar nicht	□ selten	□ gelegentlich	□ häufig
Weiteres	□ Magenschmerzen		□ Sodbrennen	

Abdomen

Aufstoßen	□ gar nicht	□ selten	□ gelegentlich	□ häufig
Übelkeit	□ gar nicht	□ selten	□ gelegentlich	□ häufig
Erbrechen	□ gar nicht	□ selten	□ gelegentlich	□ häufig
Bauchschmerzen	□ Oberbauch	□ Unterbauch	□ Generell	
Geblähter Bauch	□ gar nicht	□ selten	□ gelegentlich	□ häufig
Bauchgeräusche	□ gar nicht	□ selten	□ gelegentlich	□ häufig
Verdauungsprobleme	□ gar nicht	□ selten	□ gelegentlich	□ häufig

Appetit:

Appetitlosigkeit	□ gar nicht	□ selten	□ gelegentlich	□ häufig
Vermehrter Appetit	□ gar nicht	□ selten	□ gelegentlich	□ häufig
Essen kann nicht genossen werden	□ gar nicht	□ selten	□ gelegentlich	□ häufig

Welches Essen/Getränk mögen Sie (Mehrfachauswahl möglich):

□ süß □ salzig □ sauer □ scharf

□ fettig □ kalt □warm

□ Fleisch/Fisch □ Gemüse/Obst □ Milchprodukte □ Limonaden

Essen oder Getränke, die Sie nicht mögen (Bitte unterstreichen Sie):

Zimt, Pfeffer, Sellerie, Ingwer, japanische Jamswurzel, Sesam: ……………………………….

Essgewohnheiten:

Große Mahlzeit (Frühstück, Mittag-, Abendessen)	□ regelmäßig	□ unregelmäßig
Snacks	□ Ja	□ Nein
Snack vor dem Schlafengehen	□ Ja	□ Nein

Stuhlgewohnheiten

Häufig Obstipation/ Verstopfung	□ gar nicht	□ selten	□ gelegentlich	□ häufig
Häufig Durchfall	□ gar nicht	□ selten	□ gelegentlich	□ häufig
Abwechselnd Obstipationen und Durchfall				
	□ gar nicht	□ selten	□ gelegentlich	□ häufig
Hämorrhoiden	□ gar nicht	□ selten	□ gelegentlich	□ häufig

Urin:

Wie häufig müssen Sie innerhalb von 24 h Wasserlassen? □ 1-3 □ 4-6 □ 7-9 □ >10

Wie häufig müssen Sie in der Nacht Wasserlassen? ……………………

Urinmenge:	□ Viel	□ Normal	□ Wenig
Haben Sie Probleme beim Wasserlassen?	□ Ja	□ Nein	
Haben Sie Schmerzen beim Wasserlassen?	□ Ja	□ Nein	
Haben Sie Harninkontinenz feststellen können?	□ Ja	□ Nein	

Steifheit/Ungelenkigkeit

Nacken	□ gar nicht	□ selten	□ gelegentlich	□ häufig
Schultern	□ gar nicht	□ selten	□ gelegentlich	□ häufig
Hüften	□ gar nicht	□ selten	□ gelegentlich	□ häufig

Kältegefühl/Schüttelfrost

Hände	□ gar nicht	□ selten	□ gelegentlich	□ häufig
Füße	□ gar nicht	□ selten	□ gelegentlich	□ häufig
Po	□ gar nicht	□ selten	□ gelegentlich	□ häufig
Bauch	□ gar nicht	□ selten	□ gelegentlich	□ häufig
Rücken	□ gar nicht	□ selten	□ gelegentlich	□ häufig
Generell	□ gar nicht	□ selten	□ gelegentlich	□ häufig

Menstruationsüberblick

Ihre erste Menstruation war im Alter von: ……………… Jahren

Besteht die Wahrscheinlichkeit einer Schwangerschaft? □ Ja □ Nein

Benutzen Sie Schmerzmittel während der Periode? □ Ja □ Nein

Anzahl der Kinder: …………………..

Menopause im Alter von: ……………….. Jahren

Unregelmäßigkeiten	□ gar nicht	□ selten	□ gelegentlich	□ häufig
Monatsblutung	□ stark	□ mittelstark	□ schwach	

Reizbarkeit vor der Menstruation (PMS)

□ gar nicht □ selten □ gelegentlich □ häufig

Wie haben Sie den Fragebogen empfunden:

□ hilfreich □ weniger hilfreich □ nicht so gut

Vielen Dank für Ihre Mitarbeit!
